# Phenolics from *Ilex rotunda* Possess Antioxidative Effects and Block Activation of MAPK and NF-κB Signaling by Inhibiting IL-2 Production in CD3/CD28 Activated Jurkat T Cells

**DOI:** 10.3390/antiox14030281

**Published:** 2025-02-27

**Authors:** Ducdat Le, Vinhquang Truong, Thinhulinh Dang, Soojung Yu, Thientam Dinh, Mina Lee

**Affiliations:** 1 College of Pharmacy, Research Institute of Life and Pharmaceutical Sciences, Sunchon National University, 255 Jungangno, Suncheon 57922, Jeonnam, Republic of Korea; ddle@scnu.ac.kr (D.L.); 1243011@s.scnu.ac.kr (V.T.); 1220173@s.scnu.ac.kr (T.D.); 1243010@s.scnu.ac.kr (T.D.); 2 Department of Natural Cosmetics Science, Natural Cosmetics Research Institute, Sunchon National University, 255 Jungangno, Suncheon 57922, Jeonnam, Republic of Korea; 1223002@s.scnu.ac.kr

**Keywords:** *Ilex rotunda*, antioxidant, IL-2, MAPK, NF-κB, phenolics

## Abstract

*Ilex rotunda*, an evergreen tree in the holly family, is a traditional medicine with a high phenolic content and various pharmacological effects. This study aimed to investigate phenolic constituents from enriched fractions guided by a total phenolic assay along with a feature-based molecular network. Nine compounds were isolated and identified using multiple chromatography and spectroscopic techniques. These isolates exhibited significantly high antioxidative effects in both free radical scavenging and ROS assays. They also remarkedly alternated interleukin (IL)-2 production in CD3/CD28-stimulated Jurkat T cells. The Western blotting assay suggested that these active compounds might decrease IL-2 production by blocking the activation of NF-κB and MAPK signaling pathways by downregulating the phosphorylation of p38 and p65 proteins as well as ERK and JNK kinases. Molecular docking data confirmed the above-mentioned biological properties of those active compounds by evaluating their binding affinities for target proteins. Our findings offer guidance for assessing the potential of phenolic chemicals from *I. rotunda* as pharmacological products to improve oxidative stress and enhance immune response in more in-depth studies.

## 1. Introduction

Free radicals are characterized by their high reactivity due to the presence of unpaired electrons in their molecular structure, making them unstable and eager to react with other molecules to gain stability by pairing up their electrons. They belong to reactive oxygen species (ROS). These radicals and peroxynitrite show both positive and negative effects on the immune system [1]. They play a key regulatory role in intracellular signaling cascades and some important roles in host defense by neutralizing pathogens through respiratory burst to eliminate invading microbes or regulating the activation of immune cells by modulating the redox cascade [2,3], in which ROS can damage cellular components and lead to their destruction and recovery. ROS can activate various immune signaling pathways at low or moderate levels to regulate pro-inflammatory cytokines in order to recruit more immune cells coming into sites of injury or infection by amplifying the immune response [4]. However, excessive free radicals and ROS can have widespread detrimental effects, including DNA mutations, lipid peroxidation, and protein damage, leading to cellular dysfunction and aging. In addition, executive ROS generation is known to contribute to inflammatory conditions and immune dysfunction, leading to the development of cardiovascular diseases, neurodegenerative diseases, cancer, and autoimmune disorders [5,6]. Therefore, it is important to regulate ROS production. Maintaining a balance between oxidative stress and antioxidant defenses is essential for health.

Phenolic compounds are the most abundant heterogeneous group of secondary metabolites of plants. They play vital roles in protecting plants from diseases and environmental UV radiation [7]. These chemicals show antioxidative [8] and anti-inflammatory [9] potential. According to earlier research, phenolics also significantly influenced immune response by interacting with important molecular targets related to inflammation and immune cell functions through several signaling pathways [10,11]. Phenolics in particular have been shown to affect the synthesis of IL-2, which can either boost or suppress immunological responses that impact T cell activation, differentiation, and immune response [12]. A plant medicine called *Ilex rotunda* is used for several ailments. Previous studies have revealed that *I. rotunda* contains many phenolics possessing broad bioactivities, including anti-inflammatory, anticancer, and antidiabetic capacity [13]. Particularly, chemical components isolated from this plant possess potent antioxidative effects of caffeoylquinic acid, syringin, and phenolic glycosides [14,15]. Our primary study about this plant [16] has demonstrated that extracts and fractions of both leaves and twigs of this plant have high contents of phenolic compounds guided by feature-based molecular network (FBMN) analysis.

In our ongoing research of finding active constituents from natural sources, leaves and twigs of *I. rotunda* were investigated for their biological potential regarding active compounds having antioxidative activities toward radicals, ROS, and immune responses in CD3/CD28-activated Jurkat T cells. According to FBMN and total phenolic guidance, important materials were separated and compounds isolated were evaluated for their biological activities using in vitro, Western blotting, and in silico analyses.

## 2. Materials and Methods

### 2.1. Total Phenolic Content (TPC) Assay

The twigs and leaves of *I. rotunda* were collected from Sunchon National University (Gurye, Republic of Korea) in November 2023 and identified by Professor Mina Lee (College of Pharmacy, Sunchon National University). A voucher specimen (SCNUP 27-2024) was stored at the Pharmacognosy Laboratory, College of Pharmacy, Sunchon National University, Suncheon-si, Jeonnam-do, Republic of Korea. The TPC assay followed a previous report [17]. Briefly, total extracts of leaves and twigs along with their fractions were dissolved in DMSO solvent (2%, Sigma-Aldrich, Darmstadt, Germany) to make the stock concentration at 10,000 µg/mL. These samples were diluted 10 times with distilled water. Then, 1 mL of distilled water was added to 100 µL of each sample solution. Next, 100 µL of Folin–Ciocalteu’s phenol reagent (Sigma-Aldrich, Darmstadt, Germany) was added above each mixture. After 6 min, 1 mL of sodium carbonate (Na_2_CO_3_, 7%, Duksan, Ansan city, Gyeonggi, Republic of Korea) was added to each mixture. All the solutions were mixed after the last step and kept in the dark for 90 min. Finally, the absorbance was measured at 750 nm using a spectrophotometer. Gallic acid (Sigma-Aldrich, Bangalore, India) was used as a standard solution with a concentration range from 7.8125 to 500 µg/mL to build the calibration curves. The total phenolic content was measured in “mg gallic acid equivalents per g extracts (GAE, mg)/dried extract (DE, g)”.

### 2.2. Isolation of Compounds (***1***–***9***) from I. rotunda

Continuing our previous study [13], the methylene chloride (MC) fractions of both leaf and twig extracts, showing a high content of triterpenoid compounds, were subjected to separate the target compounds. The twig’s MC fraction was subjected to an RP-MPLC (YMC Triart ODS C_18_, 250 × 20 mm, 5.5 µm) eluted with a gradient solvent system of methanol in water (containing 0.1% formic acid) from 35% (B) to 100% (B) for 150 min, flow rate of 5.0 mL/min, and UV detection at 200, 216, and 260 nm, to obtain 14 fractions (MC_A–MC_N). Subfraction MC_L (259.6 mg) was isolated to prep HPLC using a Triart C_18_ column (10 × 250 mm, 5 μm), UV detection at wavelength 254 nm, flow rate of 3.0 mL/min, elution with a mobile phase of water (containing 0.1% formic acid, A) and acetonitrile (B) as a gradient solvent system [0 min (48% B)–10 min (60% B)–50 min (61% B)–80 min (100% B)], to obtain 2 peaks at *t*_R_ 17 min and *t*_R_ 50 min. Then, the peak at *t*_R_ 50 min was further purified by using a preparative thin layer chromatography-silica gel 60 F_254_ (1.05554.0001, Merck, Rahway, NJ, USA) with a mobile phase of methylene chloride (MC) and methanol (M) at a ratio of 15:1 to obtain compound **1**. The MC_M subfraction was subjected to a Biotage Sfar column, eluted with a gradient solvent system of ethyl acetate in *n*-hexane from 15% to 100% for 110 min, a flow rate of 5 mL/min, and UV detection at 205 and 225 nm to obtain 7 fractions (MC_M1–MC_M7). MC_M5 (34.8 mg) was subjected to preparative thin layer chromatography-silica gel 60 gel RP-18 F_254S_ plates (1.15685.0001, Merck) with a mobile phase of acetone (A) and distilled water (DW) at a ratio of 6:1 to yield compounds **2** and **4**.

The MC fraction of leaves was subjected to a Biotage Sfar C_18_ 400 g column, eluted with a gradient solvent system of methanol in water from 30% to 100% for 100 min, a flow rate of 100 mL/min, and UV detection at 200 and 254 nm, to obtain 23 fractions (MC1–MC23). Preparative HPLC accomplished subfraction MC4 (37.6 mg) on the YMC-Triart C_18_ column (10 × 250 mm, 5 µm) at a flow rate of 3.0 mL/min with a H_2_O-CH_3_CN gradient (30:70–0:100) and detection wavelength of 313 nm to yield compound **3** (*t*_R_ 45 min), compound **5** (*t*_R_ 62 min), compound **6** (*t*_R_ 49 min), and compound **7** (*t*_R_ 59 min). Furthermore, the B fraction of twigs was subjected to prep HPLC using a YMC Triart C_18_ column (10 × 250 mm, 5 µm), UV detection at wavelength 202 nm, flow rate 3.0 mL/min, and eluted with a gradient solvent system from 0 min (15% B) to 88 min (100% B), to obtain compounds **8** (*t*_R_ 55 min) and **9** (*t*_R_ 45 min).

#### Spectroscopic Data of Compounds **1**–**9**

Zhebeiresinol (**1**): White amorphous powder; HR-ESI-MS: *m*/*z* 279.0875 [M-H]^−^ (calc. for C_14_H_15_O_6_, 279.0869); ^1^H NMR (600 MHz, CD_3_OD): 3.15–3.22 (1H, m, H-1′), 4.67 (1H, d, *J* = 8.4 Hz, H-2′), 4.31 (1H, t, *J* = 8.7 Hz, H-4′), 3.54–3.57 (1H, td, *J* = 3.4, 8.8 Hz, H-5′), 4.09 (1H, dd, *J* = 3.4, 9.2 Hz, H-7′), 4.55 (1H, dd, *J* = 6.8, 9.6 Hz, H-8′), 6.67 (2H, s, H-2), 3.85 (3H, s, 3-OCH_3_, 5-OCH_3_), 6.09 (1H, brs, 4-OH), 6.67 (2H, brss, H-2, 6); ^13^C NMR (150 MHz, CD_3_OD): 131.0 (C-1), 103.0 (C-2), 148.7 (C-3), 134.1 (C-4), 148.7 (C-5), 103.0 (C-6), 46.2 (C-1′), 85.9 (C-2′), 69.9 (C-4′), 48.7 (C-5′), 178.1 (C-6′), 70.4 (C-8′), 56.1 (3, 5-OCH_3_).

Syringaresinol (**2**): White amorphous powder; HR-ESI-MS: *m*/*z* 417.1552 [M-H]^−^ (calc. for C_22_H_25_O_8_, 417.1549); ^1^H NMR (600 MHz, CD_3_OD): 4.72 (2H, d, *J* = 4.0 Hz, H-7), 4.26 (2H, dd, *J* = 6.9, 9.0 Hz, H-9a), 3.88 (2H, dd, *J* = 3.8, 9.3 Hz, H-9b), 4.72 (2H, d, *J* = 4.0 Hz, H-7′), 4.26 (2H, dd, *J* = 6.9, 9.0 Hz, H-9′a), 3.88 (2H, dd, *J* = 3.8, 9.3 Hz, H-9′b), 6.66 (4H, s, H-2, H-6), 3.14 (2H, m, H-8), 6.66 (4H, s, H-2′, H-6′), 3.14 (2H, m, H-8′), 3.84 (12H, s, 3-OCH_3_, 5-OCH3, 3′-OCH_3_, 5′-OCH_3_); ^13^C NMR (150 MHz, CD_3_OD): 133.2 (C-1), 104.6 (C-2), 149.4 (C-3), 136.3 (C-4), 149.4 (C-5), 104.6 (C-6), 87.6 (C-7), 55.5 (C-8), 72.8 (C-9), 133.2 (C-1′), 104.6 (C-2′), 149.4 (C-3′), 136.3 (C-4′), 149.4 (C-5′), 149.4 (C-5′), 104.6 (C-6′), 87.6 (C-7′), 55.5 (C-8′), 72.7 (C-9′), 56.8 (3, 5-OCH_3_, 3′, 5′-OCH_3_).

(+)-Pinoresinol (**3**): White amorphous powder; HR-ESI-MS: *m*/*z* 357.1341 [M-H]^−^ (calc. for C_20_H_21_O_6_, 357.1338); ^1^H NMR (600 MHz, CD_3_OD): 6.95 (2H, d, *J* = 2.0 Hz, H-2), 6.81 (2H, dd, *J* = 1.9, 8.1 Hz, H-5), 6.77 (2H, d, *J* = 8.1 Hz, H-6), 4.71 (2H, d, *J* = 4.4 Hz, H-7), 4.24 (2H, dd, J = 6.9, 9.1 Hz, H-9), 6.95 (2H, d, *J* = 2.0 Hz, H-2′), 6.81 (2H, dd, *J* = 1.9, 8.1 Hz, H-5′), 6.77 (2H, d, *J* = 8.1 Hz, H-6′), 4.71 (2H, d, *J* = 4.4 Hz, H-7′), 4.24 (2H, dd, *J* = 6.9, 9.1 Hz, H-9′), 3.15 (2H, m, H-8), 3.15 (2H, m, H-8′), 3.85 (6H, s, 3, 3′-OCH_3_); ^13^C NMR (150 MHz, CD_3_OD): 133.8 (C-1), 108.6 (C-2), 149.2 (C-3), 147.4 (C-4), 113.8 (C-5), 116.1 (C-6), 87.5 (C-7), 55.4 (C-8), 72.6 (C-9), 133.8 (C-1′), 108.6 (C-2′), 149.2 (C-3′), 147.4 (C-4′), 113.8 (C-5′), 116.1 (C-6′), 87.5 (C-7′), 55.4 (C-8′), 72.6 (C-9′), 56.4 (3, 3′-OCH_3_).

(+)-Medioresinol (**4**): White amorphous powder; HR-ESI-MS: *m*/*z* 387.1447 [M-H]^−^ (calc. for C_21_H_23_O_7_, 387.1444); ^1^H NMR (600 MHz, CD_3_OD): 4.69 (d, *J* = 4.3 Hz, H-7), 6.92 (d, *J* = 2.0 Hz, H-2′), 6.74 (d, *J* = 8.1 Hz, H-5′), 6.79 (dd, *J* = 2.0, 8.0 Hz, H-6′), 4.69 (d, *J* = 4.3 Hz, H-7′), 6.63 (s, H-2, 6), 3.12 (m, H-8), 3.83 (m, H-9a), 4.23 (m, H-9b), 3.12 (m, H-8′), 3.83 (m, H-9′a), 4.23 (m, H-9′b), 3.83 (s, 3, 5-OCH_3_), 3.84 (s, 3′-OCH_3_); ^13^C NMR (150 MHz, CD_3_OD): 131.2 (C-1), 103.4 (C-2), 149.3 (C-3), 135.1 (C-4), 149.3 (C-5), 103.4 (C-6), 87.1 (C-7), 54.9 (C-8), 72.8 (C-9), 131.2 (C-1′), 110.6 (C-2′), 147.2 (C-3′), 135.1 (C-4′), 115.7 (C-5′), 119.8 (C-6′), 87.1 (C-7′), 54.9 (C-8′), 72.8 (C-9′), 56.2 (3, 5-OCH_3_), 56.1 (3′-OCH_3_).

(*Z*)-2-(2,4-Dihydroxy-2,6,6-trimethylcyclohexylidene) acetic acid (**5**): White amorphous powder; HR-ESI-MS: *m*/*z* 213.1139 [M-H]^−^ (calc. for C_11_H_17_O_4_, 213.1127); ^1^H NMR (600 MHz, CD_3_OD): 2.42 (dt, *J* = 2.6, 13.3 Hz, H-3a), 1.78 (dd, *J* = 3.3, 14.2 Hz, H-3b), 4.21 (quint, *J* = 3.5 Hz, H-4), 1.99 (ddd, *J* = 2.2, 3.1, 14.4 Hz, H-5a), 1.99 (dt, *J* = 3.7, 14.4 Hz, H-5b), 5.75 (s, H-7), 1.47 (s, 6-CH_3_), 1.28 (s, 6-CH_3_), 1.76 (s, 2-CH_3_); ^13^C NMR (150 MHz, CD_3_OD): 185.4 (C-1), 88.7 (C-2), 46.5 (C-3), 67.3 (C-4), 47.9 (C-5), 36.8 (C-6), 113.3 (C-7), 171.7 (C-8), 27.4 (6-CH_3_), 31.0 (6-CH_3_), 26.6 (2-CH_3_).

Rel-(*Z*)-2-(2,4-dihydroxy-2,6,6-trimethylcyclohexylidene) acetic acid (**6**): White amorphous powder; ^1^H NMR (600 MHz, CD_3_OD): 2.42 (dt, *J* = 2.6, 13.3 Hz, H-3a), 1.78 (dd, *J* = 3.3, 14.2 Hz, H-3b), 4.21 (quint, *J* = 3.5 Hz, H-4), 1.99 (ddd, *J* = 2.2, 3.1, 14.4 Hz, H-5a), 1.99 (dt, *J* = 3.7, 14.4 Hz, H-5b), 5.75 (s, H-7), 1.47 (s, 6-CH_3_), 1.28 (s, 6-CH_3_), 1.76 (s, 2-CH_3_).

Coniferaldehyde (**7**): White amorphous powder; HR-ESI-MS: *m*/*z* 177.0563 [M-H]^−^ (calc. for C_10_H_9_O_3_, 177.0552); ^1^H NMR (600 MHz, CD_3_OD): 7.13 (1H, d, *J* = 2.3 Hz, H-2), 6.69 (1H, d, *J* = 8.4 Hz, H-5), 7.10 (1H, dd, *J* = 2.6, 8.2 Hz, H-6), 7.54 (1H, d, *J* = 15.4 Hz, H-1′), 6.52 (1H, dd, *J* = 8.1, 15.4 Hz, H-2′), 9.46 (1H, d, *J* = 8.1 Hz, H-3′), 3.85 (3H, s, 3-OCH_3_); ^13^C NMR (150 MHz, CD_3_OD): 126.7 (C-1), 112.3 (C-2), 151.7 (C-3), 149.5 (C-4), 116.7 (C-5), 125.1 (C-6), 156.2 (C-1′), 127.6 (C-2′), 196.1 (C-3′).

4-Hydroxybenzaldehyde (**8**): White powder; HR-ESI-MS: *m*/*z* 121.0299 [M-H]^−^ (calc. for C_7_H_5_O_2_, 121.0290); ^1^H NMR (600 MHz, CD_3_OD): 7.64 (2H, d, *J* = 9.2 Hz, H-2, H-6), 6.81 (2H, d, *J* = 9.3 Hz, H-3, H-5); ^13^C NMR (150 MHz, CD_3_OD): 131.2 (C-1), 132.8 (C-2), 116.2 (C-3), 165.3 (C-4), 116.2 (C-5), 132.8 (C-6), 191.9 (CHO).

*p*-Hydroxy-benzoic acid (**9**): White amorphous powder; HR-ESI-MS: *m*/*z* 137.0246 [M-H]^−^ (calc. for C_7_H_5_O_3_, 137.0239); ^1^H NMR (600 MHz, CD_3_OD): 7.92 (2H, d, *J* = 9.34 Hz, H-2, H-6), 6.43 (2H, d, *J* = 9.35 Hz, H-3, H-5); ^13^C NMR (150 MHz, CD_3_OD): 121.2 (C-1), 132.3 (C-2), 115.7 (C-3), 164.2 (C-4), 115.7 (C-5), 132.3 (C-6), 171.2 (COOH).

Analysis of the feature-based molecular network of extracts and fractions of leaves and twigs of *I. rotunda* was performed following our previous data [13] to separate the phenolics detected from the GNPS molecular network.

### 2.3. Biological Assays

#### 2.3.1. DPPH and ABTS Assays

The DPPH and ABTS radical scavenging activities were conducted by following our previous method [17]. Briefly, the capacity of each sample to scavenge the DPPH (Sigma-Aldrich, St. Louis, MO, USA) radical was used to assess its antioxidant activity. A 100-μL volume of DPPH (200 μM) in ethanol solution and 100 μL of sample volume, which had been diluted in ethanol to final concentrations (10 and 100 μg/mL), were contained in each well. Under the same conditions, a control was made by substituting ethanol for the sample amount. After carefully mixing each well, they were incubated in the shade for 30 min at room temperature before measurement at 517 nm by using a microreader (Epoch, Biotek Instruments, Winooski, VT, USA). Furthermore, ABTS (Sigma-Aldrich, Co.) was combined with 2,2′-azobis(2-aminopropane) dihydrochloride (7 mM) at a concentration of 2.45 mM, and the mixture was then allowed to react for 16 h at 4 °C. Before being applied to 96-well plates, 50 µL of the sample and 100 µL of the ABTS solution were combined and allowed to react for 20 min at room temperature. At 734 nm, the absorbance was measured. The active compounds (**1**, **2**, and **4**) were further performed at different concentrations ranging from 0 to 80 μM to determine the EC_50_ values for both DPPH and ABTS radical scavenging activity.

#### 2.3.2. Reactive Oxygen Species (ROS) Assay

##### RAW264.7 Cell Culture and Viability

The Korean Cell Lines Bank (Seoul, Republic of Korea) provided RAW264.7 cells. In a humidified atmosphere with 5% CO_2_, cells were cultivated at 37 °C and kept in Dulbecco’s modified Eagle’s medium (DMEM) supplemented with 10% heat-inactivated FBS, streptomycin sulfate (100 µg/mL), and penicillin (100 IU/mL). Following the seeding of cells onto 96-well plates, the cells were pre-incubated for 20 h; then, compounds were added for an hour, followed by stimulation with LPS (1 µg/mL) for 20 h, treatment with compounds (**1**–**9**) for 2 h, and stimulation with LPS (100 ng/mL) for 20 h. For 4 h, the cultivated cells were treated at 37 °C with MTT. Subsequently, 100 µL of dimethyl sulfoxide (Sigma Aldrich, St. Louis, MO, USA) was added after the supernatants were eliminated. Five minutes later, a microplate reader (Bio Tek Instruments, Winooski, VT, USA) was used to measure the formazan crystals’ absorbance at 570 nm.

##### Measurement of ROS Production

Compounds at concentrations of 10, 20, and 40 µM were added to cells that had been seeded in a 6-well plate at a density of 1 × 10^4^ cells/well for 20 h. After discarding all cell culture medium, 10 µM dichlorodihydrofluorescein diacetate diluted with 1/1000 in a serum-free medium was added. Following incubating for 20 min in the dark at 37 °C, the cells were rinsed 3 times with PBS. An inverted fluorescence microscope was used to view the cells, and a fluorescent microplate reader at 485 and 530 nm was used to further detect them.

#### 2.3.3. Jurkat T Cell Culture and Stimulation

Jurkat T cells were grown in RPMI medium (Gibco-RBL, Gaithersburg, MD, USA) supplemented with 10% fetal bovine serum (FBS, AusGeneX, Santa Clara, CA, USA) and PenStrep (Gibco-RBL). The cell lines were cultured at 37 °C in a humidified incubator containing 5% CO_2_ and 95% air. Jurkat T cells (1 × 10^6^) were stimulated with CD3/CD28 (7 µg/mL and 2 µg/mL CD3 and CD28, respectively) for 24 h. For the anti-CD3/CD28 stimulation, cells were added on the culture dish coated with anti-CD3 antibody. Anti-CD28 (2 μg/mL) antibody was then treated right after the addition of cells. For superantigen stimulation, T cells were incubated with staphylococcus enterotoxin E (1 μg/mL)-pulsed Raji B cells. For the pre- or post-treatment experiments, various concentrations of compounds were added 60 min before or 30 min after the stimulation of T cells.

#### 2.3.4. The Enzyme-Linked Immunosorbent Assay (Elisa) Assay

Jurkat T cells were treated as above in T cell stimulation and treatment with compounds. The supernatants were collected, and the concentrations of IL-2 were measured using the Duoset Human IL-2 ELISA Kit (R&D Systems, Minneapolis, MN, USA) according to the manufacturer’s instructions.

#### 2.3.5. Western Blot Assay

The cells were stimulated with CD3/CD28 for 2 h pretreatment. PRO-PREPTM (Intron Biotechnology, Seoul, Republic of Korea) was then used to lyse the cells, supplemented with the Pierce™ Bradford Protein Assay Kit (Thermo Fisher Scientific, Waltham, MA, USA). Using 8% and 10% acrylamide gels, proteins (20 μg) were separated by SDS-PAGE and then transferred to a PVDF membrane. The following antibodies were purchased by Cell Signaling Technology (Danvers, MA, USA): p-p38 (38 kDa), p38 (38 kDa), p-p65 (65 kDa), p65 (65 kDa), Iκαβ (36 kDa), and p-Iκαβ (36 kDa). The supplier of β-actin (AB_2289199, 42 kDa) was BD Bioscience, located in San Jose, CA, USA. ERK (44 kDa) and p-ERK (44 kDa) were acquired from Cell Signaling Technology, located in Beverly, MA, USA. Following their transfer to PVDF membranes, the blots were blocked for 2 h using 5% skim milk, then incubated for 18 h at 4 °C with the primary antibodies diluted 1:1000 in 2.5% skim milk. They were then washed 3 times with Tween20/Tris-buffered saline (T/TBS) and incubated for 2 h at room temperature with an HRP-conjugated secondary antibody diluted 1:2000 in 5% skim milk, and finally, 3 more T/TBS washes. Super Signal ™ West Femto Maximum Sensitivity Substrate (Thermo Fisher Scientific, Waltham, MA, USA) was used to detect the expression of the targeted protein.

#### 2.3.6. In Silico Studies

The crystal structures of target proteins, such as p65 (PDB ID: 1VKX), ERK (PDB ID: 6NBS), JNK (PDB ID: 1PMN), and p38 MAPK (PDB ID: 1KV2), were retrieved from the RCSB protein data bank (https://www.rcsb.org; accessed on 25 October 2024). Each protein was added to the MGL tools 1.5.6. Then, it was processed by cleaning water and heteroatoms, adding polar hydrogen atoms and Kollman charges. After the final step of receptor preparation, protein was saved in pdbqt format. The structures of compounds were downloaded from PubChem (https://pubchem.ncbi.nlm.nih.gov, 26 October 2024) in sdf formats. Subsequently, the Open Babel program (version 2.4.1) was utilized to convert them into respect pdbqt formats. After that, each structure was added to MGL tools 1.5.6 before preparation by adding Gasteiger charges. The grid box parameters were established by using Pymol program (version 4.6). The Lamarckian genetic algorithm in AutoDock 4.2.6 was applied to approach the best conformation of the ligands. The resulting complexes were established and visualized by using Discovery Studio Visualizer 2021 and Pymol programs.

#### 2.3.7. Statistical Analysis

The means ± standard deviations (S.D.) of three replicates were used to represent the data. The statistical analyses of the observed differences were conducted using the software program GraphPad Prism software version 8.0 (GraphPad Software, Inc., San Diego, CA, USA) with one-way ANOVA and Duncan’s multiple range tests. * *p* < 0.05 and ** *p* < 0.01 were considered statistically significant.

## 3. Results

### 3.1. Total Phenolic Contents of Leaves and Twigs

The Folin–Ciocalteu (FC) redox assay was employed to evaluate the total phenolic contents (TPCs) of leaf and twig samples of *I. rotunda*. The FC reagent (phosphomolybdic/phosphotungstic acid complexes) might interact with phenolic compounds in an alkaline condition by forming a blue-colored complex, which is proportional to the total phenolic content with gallic equivalent [14]. Extracts and fractions of leaves and twigs of *I. rotunda* were determined by calculating the total phenolic contents using a published method [15]. Gallic acid solution was prepared at concentrations ranging from 7.8125 µg/mL to 1000 µg/mL in triplicate. A calibration curve was built with a regression equation of Y = 0.0031x − 0.0179. The coefficient of determination (*r*^2^) was 0.9965 (Figure S1, Supplementary Materials), indicating excellent linearity for the range of concentrations studied. The total phenolic content of each sample was determined as milligram gallic acid per gram of dried extract (mg GAE/g DE). The total phenolic contents of twig samples ranged from 3.11 ± 0.01 to 7.86 ± 0.01 mg GAE/g DE. Among various fractions, the MC fraction showed the highest content of phenolics at 7.86 ± 0.01 mg GAE/g DE, followed by total extract (7.41 ± 0.05 mg GAE/g DE), E fraction (6.42 ± 0.01 mg GAE/g DE), B fraction (5.93 ± 0.01 mg GAE/g DE), and H fraction (3.11 ± 0.01 mg GAE/g DE). Leaf samples displayed TPCs ranging from 4.38 ± 0.00 to 6.36 ± 0.00 mg GAE/g DE. The MC fraction also displayed the highest TPC value of 6.36 ± 0.00 mg GAE/g DE, followed by the total extract, E, Bu, and H fractions with TPC values of 5.86 ± 0.00, 5.61 ± 0.00, 5.11 ± 0.01, and 4.38 ± 0.00 mg GAE/g DE, respectively (Figure S2, Supplementary Materials). Based on these results, both MC fractions with high TPC values were proposed for separation.

### 3.2. Molecular Network and Bioactivity Guided Isolation of Phenolics from I. rotunda

**Figure 1:**
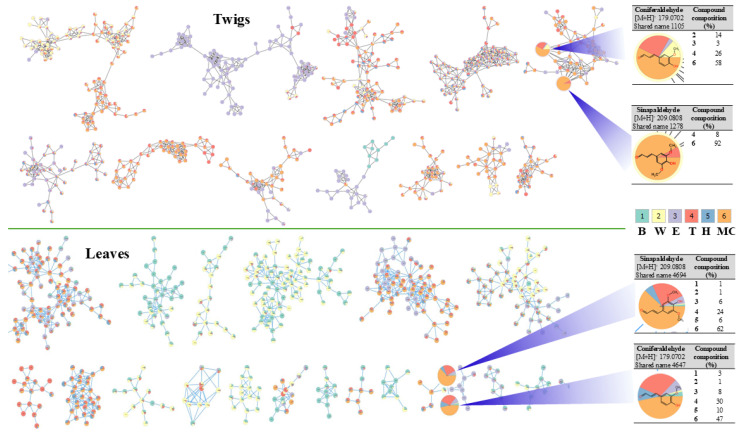
Feature-based molecular network of total extracts (T, Pink) and [Bu (B, Green), water (W, Yellow), EtOAc (E, Purple), *n*-hexane (H, Marine), and MC (MC, Orange) fractions of *I. rotunda*.

Our previous study [16] revealed that MC fractions possess potent inhibitory effects against IL-2 production in CD3/CD28-stimulated Jurkat cells. Thus, an UHPLC-Orbitrap-MS system was employed to produce HR-ESI-MS/MS data for total extracts and fractions of twigs and leaves of *I. rotunda*. Data processing was performed using the mzMine 3 program for further annotation of secondary metabolites. Their distribution patterns were determined with the FBMN-GNPS web-platform. The identification of metabolites was accelerated by analyzing their mass data dereplication of ion precursors, mass isotopic patterns, fragmentations, and spectral matching to those reported from public mass databanks along with optimization from GNPS open webtools. Among phenolic compounds annotated from clusters of the FBMN network (Figure 1), a node with a shared name of 1278 showed precursor ion peaks detected at *m*/*z* 179.0703 [M+H]^+^ from the FBMN of twig samples. It also produced fragments at *m*/*z* 148.0977 [M+H-CH_3_O]^+^ and 119.0897 [M+H-CH_3_O-CHO]^+^. The above fragmentation allowed us to assign this peak to coniferaldehyde by analyzing mass data along with data in a previous study [16]. Another peak also observed for a shared number of 1278, showing a precursor ion peak at *m*/*z* 209.0808 [M+H]^+^. This compound might produce daughter ion peaks at *m*/*z* 191.0709 [M+H-OH]^+^ and 177.0608 [M+H-OH-CH_3_]^+^, corresponding to reduction of a water molecule and a methyl group, respectively. Therefore, this peak was identified as sinapaldehyde [17]. Both phenolics were also observed in the FBMN of leaf extracts and fractions undergoing the same fragmentation pathway. Notably, both phenolic compounds displayed the highest abundance from MC fractions of both leaves and twigs. Based on these results, MC fractions of both leaves and twigs were subjected to further separation.

### 3.3. Structural Elucidation of Compounds (***1***–***9***) Isolated from Leaf and Twig Extracts of I. rotunda

**Figure 2:**
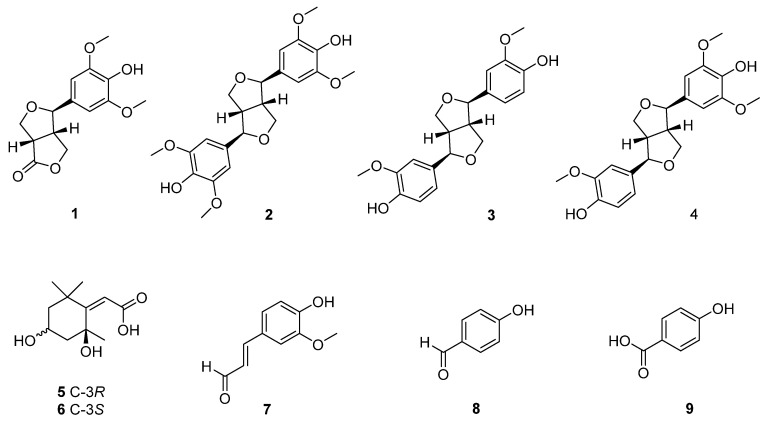
Chemical structures of compounds (**1**–**9**) isolated from MC fractions of *I. rotunda*.

Nine compounds (**1**–**9**) were isolated using multiple chromatographic techniques. The structures of these compounds were established (Figure 2) by analyzing their spectroscopic data and interpreting the fragmentation of mass data compared to those reported in the literature.

Compound **1** was isolated as a white amorphous powder. The HR-ESI-MS spectrum of **1** showed a deprotonated ion peak at *m*/*z* 279.0875 [M-H]^−^ (calc. for C_14_H_15_O_6_, 279.0869). ^1^H NMR spectrum of **1** showed a symmetric proton for a meta-substituted benzene ring at *δ*_H_ 6.67 (2H, s, H-2, 6) and two methoxy groups at *δ*_H_ 3.85 (6H, s, 3-OCH_3_, 5-OCH_3_). The presence of a furofuran moiety was established using the COSY spectrum, showing sequence correlations of H-7/H-8/H-9 and those of H-7′/H-8′/H-9′. Thus, this compound was identified as zhebeiresinol by analyzing mass and spectroscopic data compared to those reported in the literature [18].

Compound **2** was obtained as a white amorphous powder. Its HR-ESI-MS displayed a deprotonated ion peak at *m*/*z* 417.1552 [M-H]^−^ (calc. for C_22_H_25_O_8_, 417.1549). Its ^1^H and ^13^C NMR spectra showed a symmetrical structure with two meta-substituted benzene rings at *δ*_H_ 6.67 (4H, s, H-2, 6, 2′, 6′) and four groups at *δ*_H_ 3.84 (12H, s, 3-OCH_3_, 5-OCH_3_, 3′-OCH_3_, 5′-OCH_3_). The presence of a furofuran moiety was determined by the COSY spectrum, showing correlations between H-7/H-8/H-9 and those of H-7′/H-8′/H-9′. Furthermore, the HMBC spectrum showed correlations from H-2/H-6 (*δ*_H_ 6.66) to C-7/C-7′ (*δ*_C_ 87.6), confirming the partial structure of **2**. HMBC cross-peaks of methoxy groups (*δ*_H_ 3.84) to oxygenated-quaternary carbon at *δ*_C_ 149.4 confirmed the attachment of methoxy functional groups at 3-OCH_3_, 5-OCH_3_, 3′-OCH_3_, and 5′-OCH_3_. Finally, the structure of **2** was established as syringaresinol [19].

Compound **3** was isolated as a white amorphous powder. Its HR-ESI-MS displayed a deprotonated ion peak at *m*/*z* 357.1341 [M-H]^−^ (calc. for C_20_H_21_O_6_, 357.1338). ^1^H and ^13^C NMR spectra of **3** were similar to those of **2** with the reduction of two methoxy groups. Indeed, the ^1^H NMR spectrum of **3** showed a symmetrical structure with two ABX spin systems at *δ*_H_ 6.95 (2H, d, *J* = 2.0 Hz, H-2, 2′), 6.81 (2H, dd, *J* = 1.9, 8.1 Hz, H-5, 5′), and 6.77 (2H, d, *J* = 8.1 Hz, H-6, 6′) with furofuran signals at *δ*_H_ 4.71 (2H, d, *J* = 4.4 Hz, H-7, 7′), 4.24 (2H, dd, *J* = 6.9, 9.1 Hz, H-9a, 9′a), 3.85 (2H, m, H-9b, 9′b), and 3.15 (2H, m, H-8, 8′), along with two methoxy groups at *δ*_H_ 3.85 (6H, s, 3-OCH_3_, 3′-OCH_3_). The structure of **3** was determined as (+)-pinoresinol based on comparison with those reported from the literature [19].

Compound **4** was collected as a white amorphous powder. Its HR-ESI-MS displayed a deprotonated ion peak at *m*/*z* 387.1447 [M-H]^−^ (calc. for C_21_H_23_O_7_, 387.1444). ^1^H NMR spectrum of **4** demonstrated signals of an ABX spin system at *δ*_H_ 6.92 (1H, d, *J* = 2.0 Hz, H-2′), 6.79 (1H, dd, *J* = 2.0, 8.0 Hz, H-6′), and 6.74 (1H, d, *J* = 8.1 Hz, H-5′) with two meta-substituted benzene rings at *δ*_H_ 6.63 (2H, s, H-2, 6) and three methoxy groups at *δ*_H_ 3.83 (6H, s, 3-OCH_3_, 5-OCH_3_) and 3.84 (3H, s, 3′-OCH_3_), supporting the reduction of an methoxy group compared to those of **2**. The structure of **4** was determined as (+)-medioresinol by comparing ^1^H and ^13^C NMR spectra with those reported in the literature [20].

Compound **5** was isolated as a white amorphous powder. Its HR-ESI-MS displayed a deprotonated ion peak at *m*/*z* 213.1139 [M-H]^−^ (calc. for C_11_H_17_O_4_, 213.1127). ^1^H NMR spectrum of **5** displayed an olefinic proton at *δ*_H_ 5.75 (1H, s, H-7), an oxymethine signal at *δ*_H_ 4.21 (1H, quint, *J* = 3.5 Hz, H-3), two methylene groups with four protons at *δ*_H_ 2.42 (1H, dt, *J* = 2.6, 13.3 Hz, H-5a), 1.99 (1H, ddd, *J* = 2.2, 3.1, 14.4 Hz, H-3a), 1.99 (1H, dt, *J* = 3.7, 14.4 Hz, H-5b), and 1.78 (1H, dd, *J* = 3.3, 14.2 Hz, H-3b), and three methyl groups at *δ*_H_ 1.76 (3H, s, CH_3_-2), 1.47 (3H, s, 6-CH_3_), and 1.28 (3H, s, 6-CH_3_). The ^13^C NMR spectrum of **5** displayed 11 carbons, including a carbonyl group at *δ*_C_ 171.7 (C-8), two olefinic signals at *δ*_C_ 185.4 (C-1) and 113.8 (C-7), two oxygenated carbons at *δ*_C_ 88.7 (C-2) and 67.3 (C-4), two methylene carbons at *δ*_C_ 47.9 (C-5) and 46.5 (C-3), and three methyl carbons at *δ*_C_ 31.0, 27.4, and 26.6. The HMBC spectrum showed correlations of 2-CH_3_ and 6-CH_3_ to C-1, confirming the double bond Δ^1(7)^. HMBC showed cross-peaks of H-7 to C-2/C-6/C-8, suggesting an 8-COOH position. Therefore, the structure of **5** was identified as (*Z*)-2-(2,4-dihydroxy-2,6,6-trimethylcyclohexylidene) acetic acid [21].

Compound **6** was isolated as a white amorphous powder. Its spectrometry and spectroscopic data were similar to those of **5** except for the C-3 position. Thus, the structure of **6** was determined as an epimer of **5** and named rel-(*Z*)-2-(2,4-dihydroxy-2,6,6-trimethylcyclohexylidene) acetic acid [22].

Compound **7** was collected as a white amorphous powder. Its HR-ESI-MS displayed a deprotonated ion peak at *m*/*z* 177.0563 [M-H]^−^ (calc. for C_10_H_9_O_3_, 177.0552). The ^1^H NMR spectrum of **7** revealed a aldehyde proton at *δ*_H_ 9.46 (1H, d, *J* = 8.1 Hz, H-3′), two characteristic trans-olefinic protons at *δ*_H_ 7.54 (1H, d, *J* = 15.4 Hz, H-1′) and 6.52 (1H, dd, *J* = 8.1, 15.4 Hz, H-2′), and an ABX spin system at *δ*_H_ 7.13 (1H, d, *J* = 2.3 Hz, H-2), 7.10 (1H, dd, *J* = 2.6, 8.2 Hz, H-6), and 6.69 (1H, d, *J* = 8.4 Hz, H-5). Additionally, the ^13^C NMR spectrum of **7** showed a carbonyl group at *δ*_C_ 191.9 (C-3′), five unsaturated hydrocarbons at *δ*_C_ 112.3 (C-2), 116.7 (C-5), 125.1 (C-6), 156.2 (C-1′), and 127.6 (C-2′), along with three oxygenated quaternary carbons at *δ*_C_ 126.7 (C-1), 151.7 (C-3), and 149.5 (C-4). The structure of **7** was determined as coniferaldehyde by analyzing its spectroscopic data compared to those reported in the literature [22].

Compound **8** was obtained as an amorphous powder. Its HR-ESI-MS displayed a deprotonated ion peak at *m*/*z* 121.0299 [M-H]^−^ (calc. for C_7_H_5_O_2_, 121.0290). The ^1^H NMR spectrum of **8** revealed an aldehyde proton at *δ*_H_ 8.55 (1H, s, H-7) with an A_2_B_2_ spin system at *δ*_H_ 7.95 (2H, d, *J* = 9.0 Hz, H-2, 6) and 6.45 (2H, d, *J* = 9.0 Hz, H-3, 5). Thus, compound **8** was identified as 4-hydroxybenzaldehyde [23].

Compound **9** showed a structure similar to **8** by replacing an aldehyde group with a carboxylic acid group. Indeed, its HR-ESI-MS showed a deprotonated ion peak at *m*/*z* 137.0246 [M-H]^−^ (calc. for C_7_H_5_O_3_, 137.0239). Its ^1^H NMR spectrum displayed a symmetrical structure showing an A_2_B_2_ spin system at *δ*_H_ 7.88 (2H, d, *J* = 8.9 Hz, H-2, 6) and 6.82 (2H, d, *J* = 8.9 Hz, H-3, 5). Its ^13^C NMR spectrum showed a carbonyl group at *δ*_C_ 170.1 (C-7), two quaternary carbons at *δ*_C_ 163.4 (C-4) and 131.1 (C-1), and four methine carbons at *δ*_C_ 133.0 (C-2, 6) and 116.0 (C-3,5). Thus, the structure of **9** was identified as 4-hydroxybenzoic acid [24].

### 3.4. Antioxidative Effects of Isolated Compounds

**Figure 3:**
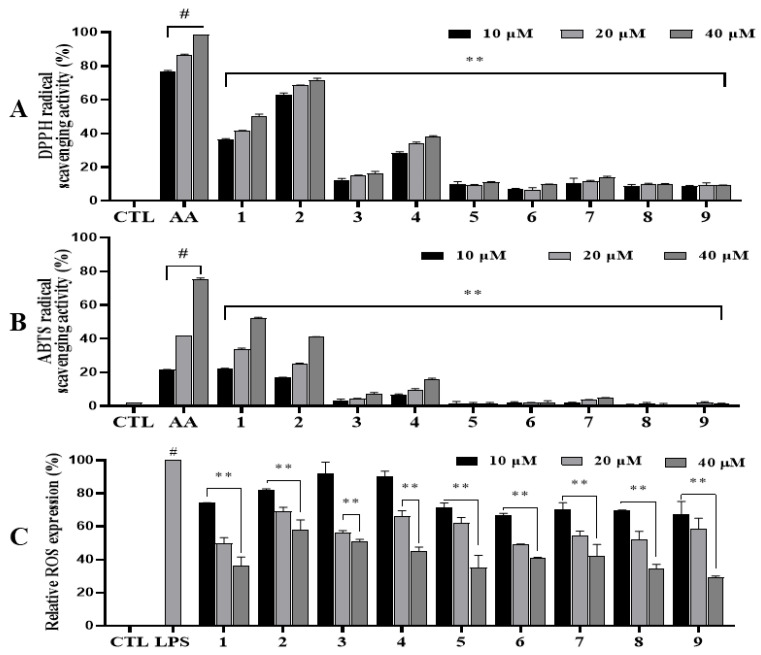
Antioxidant effect of compounds (**1**–**9**). DPPH (**A**), ABTS (**B**), and ROS (**C**) assays were performed in triplicate. The data are represented as mean ± SD. ^#^ *p* < 0.05 vs. non-treated group (CTL). ** *p* < 0.01, statistically significant compared to positive control (ascorbic acid, AA) or LPS treatment without compound addition (LPS).

The antioxidative effects of isolated compounds were evaluated through screening their abilities to scavenge DPPH and ABTS radicals. The radical scavenging activity was evaluated by the percentage decrease in absorbance of the DPPH/ABTS solution [25]. A higher inhibition rate signifies stronger antioxidant activity. At 40 µM, compounds **1** and **2** demonstrated strong radical scavenging activities toward the DPPH radical with inhibition activity of 50.01 ± 1.13% and 70.45 ± 1.06%, respectively, while compound **4** moderately scavenged the DPPH radical with an inhibition activity of 37.36 ± 0.60%. At 10 and 20 µM, compound **2** still strongly scavenged the DPPH radical with inhibition activity of 63.11 ± 0.76% and 68.42 ± 0.32%, respectively. Compounds **1** and **4** showed significant DPPH radical scavenging activities, with inhibition activities ranging from 28.32 ± 0.66% to 41.63 ± 0.13%. Other compounds showed weak DPPH radical scavenging activities at the same tested conditions (Figure 3A). ABTS is a water-soluble radical with a characteristic green-blue color (oxidized form, ABTS^+^). Its color might change from the above color to green or colorless depending on the presence of antioxidants. We found some reduction in the green-blue color with or without (negative control) the treatment of compounds. Upon treatment, compounds **1**, **2**, and **4** significantly reduced the ABTS radical at tested conditions. At 40 µM, compounds **1** and **2** strongly decreased ABTS radical formation with scavenging rates of 52.33 ± 0.30% and 40.94 ± 0.30%, respectively, compared to the negative control. Compound **4** showed some radical scavenging activity with a scavenging rate of 16.04 ± 0.38%. At both concentrations of 10 and 20 µM, compounds **1** and **2** also displayed radical scavenging activities with inhibition activities ranging from 17.15 ± 0.04% to 33.81 ± 0.52%. Other compounds showed weak or no ability to scavenge the ABTS radical at the same tested conditions (Figure 3B). Furthermore, the active compounds (**1**, **2**, and **4**) were assayed to determine their activity toward radicals. As a result, compound **2** exhibited the lowest EC_50_ value of 9.72 ± 1.99 µM, followed by **1** (EC_50_ = 39.16 ± 1.04 µM), and **4** (EC_50_ = 70.73 ± 9.21 µM) to DPPH scavenging activity. Moreover, compound **1** showed significant effect to ABST radical scavenging activity with an EC_50_ value of 33.23 ± 0.66 µM to ABTS scavenging activity, followed by 2 (EC_50_ = 42.90 ± 2.12 µM) and **4** (EC_50_ = 57.62 ± 6.00 µM) (Table S1, Supplementary Materials).

To investigate the antioxidative effects of isolated compounds, RAW264.7 cells were used to evaluate ROS production regulated by LPS stimulation. At first, RAW264.7 cells were treated with each compound to evaluate the cytotoxic effects of compounds on cell viability evaluated with the MTT assay [17]. The results indicated that these compounds did not affect cell viability at concentrations up to 40 µM. These compounds were then evaluated for their antioxidative effects by measuring ROS production induced by LPS in RAW264.70 cells. After treatment with LPS, ROS production was increased. At 40 µM, compounds **1**, **5**, **8**, and **9** inhibited ROS production at rates of 63.52 ± 4.12%, 64.97 ± 6.15%, 65.69 ± 2.31%, and 71.54 ± 0.54%, respectively (Figure 3C). At 20 µM, compounds **1**, **6**, and **8** significantly reduced ROS production induced by LPS in RAW264.7 cells. At 10 µM, compounds **1** and **5**–**9** decreased ROS production compared to control (LPS-treated cells without compound addition). No compounds showed any toxic effects on the viability of RAW264.7 cells under the tested conditions.

### 3.5. Inhibitory Effects of Isolated Compounds on CD3/CD28-Induced IL-2 Production in Activated Jurkat T Cells

**Figure 4:**
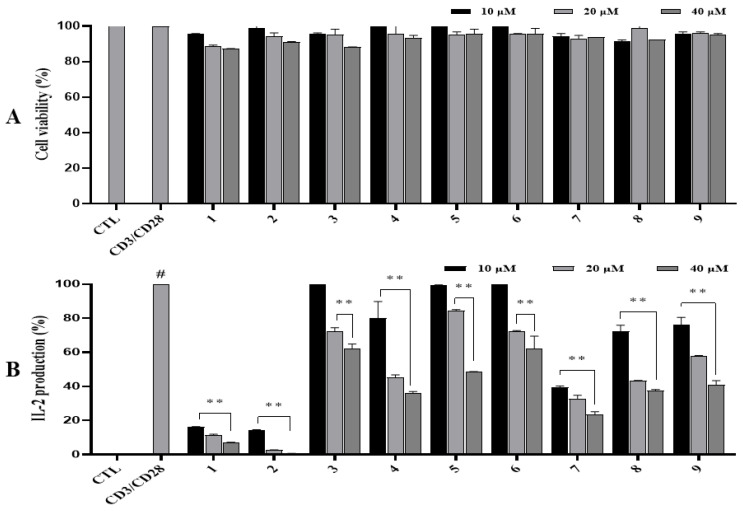
Cytotoxic (**A**) and IL-2 (**B**) production inhibitory effects of isolated compounds (**1**–**9** at 10, 20, and 40 µM) in CD3/CD28-stimulated Jurkat T cells. Cells were treated with compounds **1**–**9** (10, 20, and 40 µM) for 1 h and stimulated with CD3/CD28 (7 µg/mL and 2 µg/mL CD3 and CD28, respectively) for 24 h. IL-2 production in the culture media was quantified using an enzyme-linked immunosorbent assay (ELISA) kit. ^#^ *p* < 0.05 vs. non-treated group (CTL). Differences were significant at ** *p* < 0.01 compared to the non-sample control in CD3-CD28 stimulation.

At first, no tested compounds affected cell viability at concentrations up to 40 µM (Figure 4A). As a result, all compounds were also assessed for their inhibitory effects on CD3/CD28-induced IL-2 production in Jurkat T cells. Upon treatment with co-stimulator CD3/CD28, IL-2 secretion was significantly increased compared to the non-treated control. However, IL-2 production was regulated by compounds in a dose-dependent manner. Particularly, Compounds **1** and **2** strongly decreased IL-2 secretion induced by the co-stimulator in Jurkat cells at all tested concentrations. At a concentration of 40 µM, compound **2** potently decreased IL-2 production by approximately 99.5% compared to co-simulator without adding compound **2**. Compound **1** remarkedly inhibited IL-2 production with an inhibition rate of 97.17%. Compound **7** significantly reduced IL-2 production with an inhibition rate of 76.54%. Compounds **4**, **5**, **8**, and **9** moderately inhibited IL-2 production with inhibition rates of 64.16%, 51.61%, 62.35%, and 59.10%, respectively. At concentration of 20 µM, compound **2** markedly decreased IL-2 production with an inhibition rate of 96.54%, while compound **1** strongly decreased IL-2 production at 88.58%. Compounds **4**, **7**, and **8** significantly inhibited IL-2 production with inhibition rates of 54.66%, 67.54%, and 56.76%, respectively. At 10 µM, compounds **1** and **2** strongly reduced IL-2 production with inhibition rates of 83.85% and 85.86%, respectively. Compound **7** significantly decreased IL-2 production with an inhibition rate of 60.70%. Other compounds (**3** and **6**) showed weak effects on CD3/CD28-induced IL-2 production in activated Jurkat T cells (Figure 4B).

### 3.6. Compounds ***1***, ***2***, and ***7*** Inhibit IL-2 Production by Interfering with MAPK Activation

**Figure 5:**
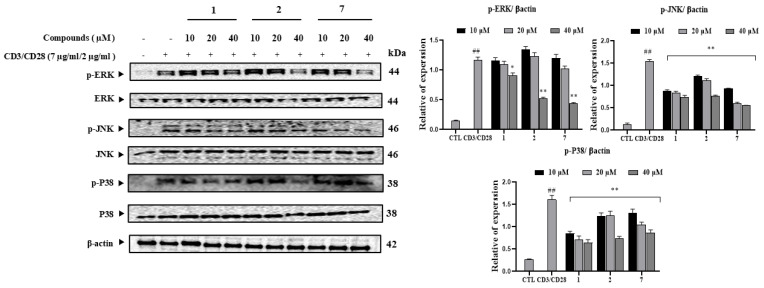
The inhibitory effects of isolated compounds (**1**–**9**) on the CD3/CD28-induced MAPK signaling pathway in Jurkat T cells. After pretreating cells with the compounds (10, 20, and 40 μM) for 1 h, cells were stimulated with CD3/CD28 for 15 min. Total proteins were then isolated, separated by SDS-PAGE, and immunoblotted using specific antibodies for p-ERK, p-JNK, p-p38, ERK, JNK, and p38. β-Actin served as an internal control. The relative optical density ratio vs. β-actin or total form was determined using a densitometric analysis program (Bio-Rad Quantity One Software, version 4.6.3 (Basic), Bio-Rad Laboratories Inc., CA, USA) normalized to the internal control. ^##^ *p* < 0.05 vs. non-treated group (CTL). Differences were significant at * *p* < 0.05 and ** *p* < 0.01 compared to the control in CD3/CD28 stimulation.

To evaluate correlations between active compounds and MAPK-specific proteins, including ERK, JNK, and p38, protein expression levels were determined using Western blotting (Figure 5). Upon treatment with compounds, expression levels of ERK, JNK, and p38 proteins were upregulated. These compounds also upregulated the phosphorylation levels of ERK, JNK, and p38 proteins in a dose-dependent manner. The expression level of p-ERK was increased after CD3/CD28 simulation. At 40 µM, compounds **2** and **7** reduced the phosphorylation levels of ERK more than compound **1**. At 20 µM, compound **7** displayed a stronger inhibition on ERK phosphorylation than compounds **1** and **2**. The expression level of p-JNK was increased after CD3/CD28 simulation. When these compounds were used for treatment to determine their effects on p-JNK, it was revealed that compound **7** decreased the phosphorylation of JNK the strongest at both concentrations of 20 and 40 µM. Compounds **1** and **2** significantly decreased the phosphorylation of JNK at 40 µM. Compound **1** resulted in a stronger decrease of p-JNK expression level than compound **2**. At 10 µM, compound **1** inhibited p-JNK expression level the strongest among the three compounds. The phosphorylation of p38 was increased after CD3/CD28 stimulation. After treatment with each compound, the phosphorylation of p38 was regulated. At all tested concentrations, compound **1** suppressed the p38 phosphorylation level more than compounds **2** and **7**. At 40 µM, compound **2** inhibited the p38 phosphorylation level more than compound **7**. However, compound **7** showed a stronger suppression of p38 phosphorylation than compound **2** at 20 µM. None of the compounds tested affected β-actin expression levels under the tested conditions.

### 3.7. Compounds ***1***, ***2***, and ***7*** Inhibit IL-2 Production by Interfering with NFκB Activation

**Figure 6:**
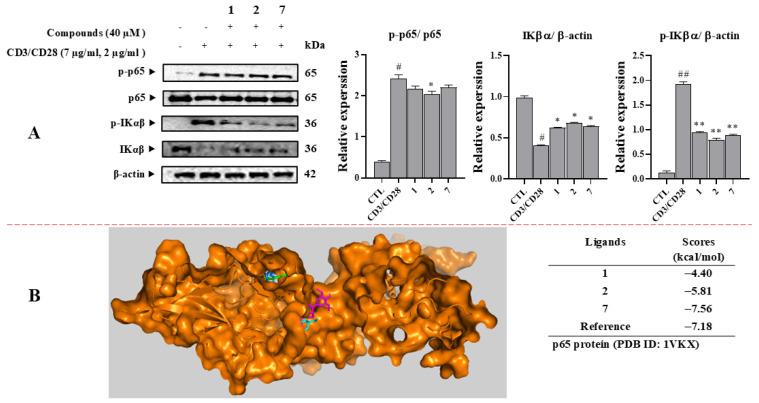
(**A**) Inhibitory effect of compounds (**1**, **2**, and **7**) in the CD3/CD28-induced NF-κB signaling pathway in Jurkat T cells. After pretreating cells with compounds (40 μM) for 1 h, cells were stimulated with CD3/CD28 for 15 min. Total proteins were then isolated, separated by SDS-PAGE, and immunoblotted using specific p-p65, p-IKαβ, p65, and IKαβ antibodies. β-Actin was applied as an internal control. Each experiment was independently performed in triplicate. The relative optical density ratio vs. β-actin or total form was determined using a densitometric analysis program (Bio-Rad Quantity One Software, version 4.6.3 (Basic), Bio-Rad Laboratories Inc., Hercules, CA, USA), normalized to the internal control. The values are expressed as the average ± SD for the three individual experiments. ^#^ *p* < 0.05, ^##^ *p* < 0.05 vs. the control group; * *p* < 0.05, ** *p* < 0.01 vs. CD3/CD28-stimulated T cells. (**B**) Molecular docking analysis of compounds **1** (Blue), **2** (Magenta), **7** (Green), docked into p65 protein.

The mechanism involved in the inhibitory effect of active compounds on IL-2 production was then investigated by examining the phosphorylation of p65 NFκB and IKαβ in signaling pathways (Figure 6A). The phosphorylation levels of p65 were increased after CD3/CD28 stimulation, indicating the activation of the NFκB pathway. However, upon treatment with active compounds at 40 µM, the p-p65 expression level was decreased, with compound **2** decreasing the p-p65 expression level more than compounds **1** and **7**.

Under normal conditions, the IκB protein can bind to the p65 subunit of NFκB, forming a complex that keeps the NFκB molecule sequestered in the cytoplasm, preventing translocation to the nucleus and the activation of gene expression. The activation of NFκB would result in IκB degradation [26]. Since compounds **1**, **2**, and **7** showed inhibitory effects on p-p65 expression, their ability to regulate IκB degradation was then examined by evaluating their effects on IKαβ pathway. The phosphorylation level of IKαβ was increased after CD3/CD28 stimulation. However, upon treatment with active compounds, such an increase in the phosphorylation level of IKαβ was decreased, with compound **2** decreasing IKαβ phosphorylation more than compounds **1** and **7**. These compounds did not affect β-actin expression under the tested conditions (Figure 6A).

### 3.8. In Silico Molecular Docking Analysis

**Figure 7:**
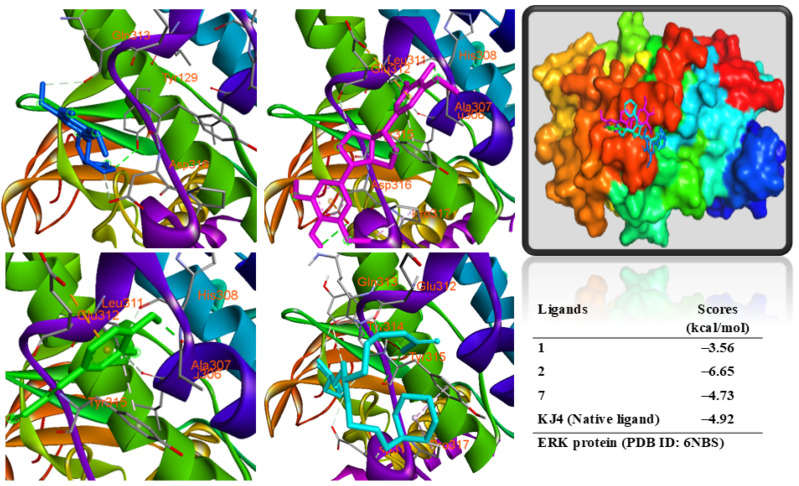
Interactions of compounds, **1** (Blue), **2** (Magenta), **7** (Green), and KJ4 (N-{3-[(2Z,4S)-1-(2-{[2-(2-amino-1H-imidazol-1-yl)ethyl](methyl)amino}ethyl)-3-(3-cyclohexylpropyl)-2-iminoimidazolidin-4-yl]propyl}guanidine, Cyan), with amino acids when they were docked into the ERK protein (PDB ID: 6NBS) from MAPK.

To estimate the binding affinity of active compounds for target proteins, molecular docking in silico was performed. Compounds (**1**, **2**, and **7**) were prepared as ligands to dock into ERK protein (Figure 7). All compounds were docked into the same region of the re-docked native ligand, indicating that these compounds were docked into the binding pose of the protein. The data demonstrated that compound **2** had a binding free energy (ΔG = −6.65 kcal/mol) lower than those of a co-crystal structure (ΔG = −4.92 kcal/mol). Compound **7** showed a similar binding free energy (ΔG = −4.73 kcal/mol) to a native ligand, while compound **1** displayed a higher binding free energy (ΔG = −3.56 kcal/mol). These compounds were occupied in the binding pose of the protein surrounded by interacting amino acids. Both compounds **2** and **7** might interact with ALA307 residue through a conventional hydrogen bond by interacting with the hydroxyl groups of ligands. Additionally, compound **2** showed a further hydrogen bond with the ASP316 amino acid and hydrophobic interactions with GLU312, TYR315, ASP316, and PRO317 residues of the protein. Compound **7** displayed hydrophobic interactions with GLU312, TYR315, and ASP316 residues. Compound **1** showed a hydrogen bond with TYR129 and other hydrophobic interactions with the ARG133, GLN313, TYR315, ASP316, and ASP319 amino acids. Therefore, ALA307, GLN312, TUR314, ASP316, and PRO317 amino acids are key residues of the binding pose of the protein (Figure S3, Supplementary Materials).

**Figure 8:**
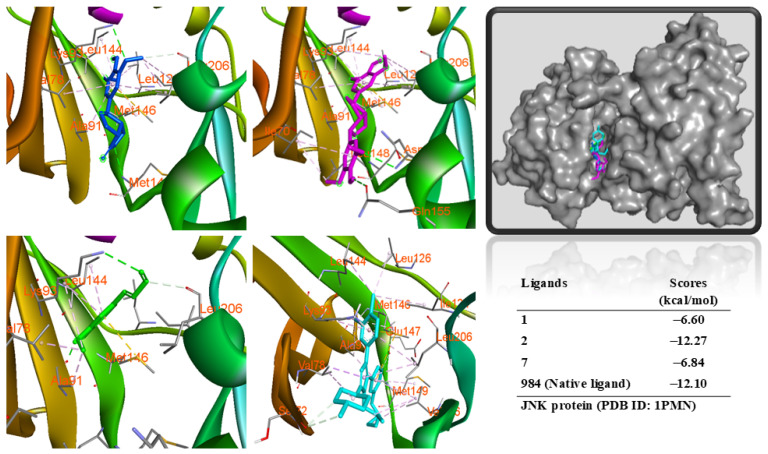
Interactions of compounds **1** (Blue), **2** (Magenta), **7** (Green), and 984 (Cyclopropyl-{4-[5-(3,4-dichlorophenyl)-2-[(1-methyl)-piperidin]-4-yl-3-propyl-3H-imidazol-4-yl]-pyrimidin-2-yl}amine, Cyan) with the amino acid when they were docked into the JNK protein (PDB ID: 1PMN) from MAPK.

Similarly, these compounds docked into the same region of the active region of the JNK protein (Figure 8). Compound **2** showed a binding free energy (ΔG = −12.27 kcal/mol) similar to a redocked native ligand (ΔG = −12.10 kcal/mol). Compounds **1** and **7** displayed a significant binding affinity to protein. All three compounds could interact with the MET146 residue of the protein through a π-sulfur interaction. In contrast, compound **2** showed two hydrogen bonds with ASN152 and GLN155 residues and important interactions with VAL78, ALA91, LYS93, LEU144, MET149, VAL196, and LEU206 residues of the binding pose of the protein. Compound **1** showed hydrogen bonds with the LYS93 and MET149 amino acids. Compound **7** demonstrated two hydrogen bonds with the ALA91 and LYS93 amino acids. Both **1** and **7** also interacted with the above hydrophobic protein (Figure S4, Supplementary Materials).

**Figure 9:**
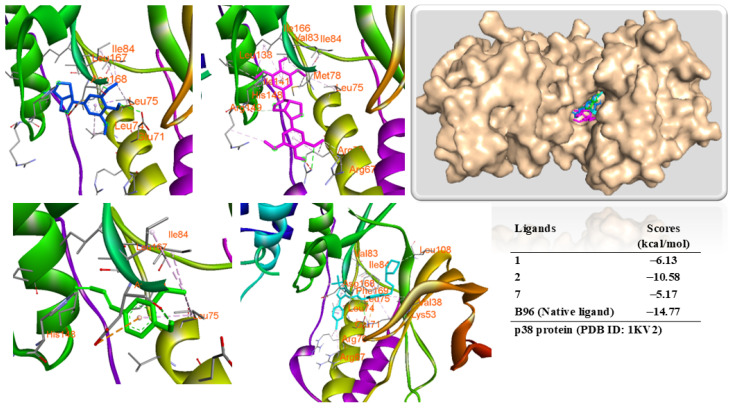
Interactions of compounds, **1** (Blue), **2** (Magenta), **7** (Green), and B96 [1-(5-tert-butyl-2-p-tolyl-2H-pyrazol-3-yl)-3-(4-(2-morpholin-4-yl-ethoxy)-naphtalen-1-yl)-urea, Cyan] with the amino acid when they were docked into the p38 protein (PDB ID: 1KV2) from MAPK.

Compounds **1**, **2**, and **7** also displayed a significant binding affinity when they were docked into the p38 protein (Figure 9). These compounds and the co-crystal structure were occupied at the same allosteric site of the binding pose of the protein. This observation indicated that the docking protocol was appropriate. Compound **2** showed a binding free energy (ΔG = −10.58 kcal/mol) lower than compounds **1** and **7** (binding free energy, ΔG = −6.13 and −5.17 kcal/mol, respectively). Compound **1** showed two hydrogen bonds with GLU71 and ASP168 residues by interacting with hydroxyl and oxygenated bonding. Compound **2** showed hydrogen bonds with ARG70 and ILE166 residues through interacting with the hydroxyl group. Compound **7** interacted with the protein through HIS148 and ASP168 by bonding with hydroxy and carboxyl groups (Figure S5, Supplementary Materials). These compounds also showed hydrophobic interactions with amino acids, including LYS53, ARG67, LEU75, ILE84, and LEU167 [27].

When these compounds were docked into the p65 protein, they showed a significant binding affinity (Figure 6B). Among them, compound **7** showed the lowest binding free energy (ΔG = −7.56 kcal/mol), followed by compounds **2** and **1** (ΔG = −5.81 and −4.11 kcal/mol, respectively). However, compounds **1** and **7** occupied the same region. They were in different locations from compounds **2** and the reference (Figure S6, Supplementary Materials). Compound **1** showed hydrogen bonds with ARG35 through an oxygenated interaction. Compound **2** interacted with key amino acids ARG187 and LEU154 and ASN155 residues through hydrogen and oxygenate-forming hydrogen bonds. Compound **7** displayed two hydrogen bonds with key amino acids ASN155 and ARG187 through the hydroxy group, along with hydrophobic interactions with ARG35 by a Van der Waals interaction [28].

## 4. Discussion

The TPC method was used to assess the phenolic contents in enriched MC fractions from both twigs and leaves of *I. rotunda*. FBMN-GNPS guidance was applied to identify phenolic potential by identifying two markers, coniferaldehyde and sinapaldehyde, which were highly expressed in MC fractions compared to other fractions based on relative quantitation. Therefore, both MC fractions were selected as potential materials for further separation from leaves and twigs of *I. rotunda*. These target fractions were separated by performing multiple chromatographic technology, resulting in the isolation of nine compounds (**1**–**9**). Their structures were determined by analyzing their spectroscopic data compared to those reported in the literature. These isolated compounds exhibited strong antioxidative effects against DPPH and ABTS radicals. They decreased ROS production in a dose-dependent manner when concentrations of 10, 20, and 40 µM were used for testing. Among the isolated compounds, compounds **1** and **2** strongly inhibited both radicals at the tested concentrations. Compound **4** moderately scavenged DPPH radicals at the tested concentrations. Based on the radical scavenging activities of these isolated compounds, a structural–activity relationship was deduced from the above antioxidative effect for compounds **2**–**4** (furan lignan). However, compound **2** showed the most potent radical scavenging activity, followed by compounds **4** and **3**. This observation indicated that increasing methoxy groups attached to an aromatic ring could gradually enhance the radical scavenging ability. It is proposed that methoxy groups could contribute electron density to the aromatic ring via both induction and resonance. Therefore, the molecular ring is reactive to electrophiles by increasing its electron density, especially at ortho and para locations [29]. This makes the molecule more polarizable and improves its ability to donate electrons to free radicals. This molecule might also stabilize free radicals, increasing its antioxidative potential and enhancing its capacity to scavenge radicals. Additionally, these compounds were further tested for their antioxidative potential toward ROS production induced by LPS-stimulated RAW264.7 cells. Data showed that all compounds significantly decreased ROS production at 40 µM. They also reduced ROS production at 20 µM.

Excessive and prolonged ROS generation is known to negatively affect T cell function throughout its activation and differentiation. ROS may hinder proper immune function and/or lead to DNA damage, protein oxidation, lipid peroxidation, mitochondrial dysfunction, and impaired immune responses. These negative effects can contribute to immune cell dysfunction, chronic inflammation, autoimmune diseases, and cancer development [30]. Therefore, maintaining a balance between ROS production and antioxidant defenses is essential for optimal immune function. Strategies that can reduce oxidative stress may help support immune health [31]. IL-2 is essential for controlling immune responses, especially for T cell activation and proliferation. The positive role of IL-2 in immune response indicates that its signaling causes T cells to develop into short-lived, terminally differentiated effector cells and stimulates immune-activated CD8+ T cells to produce important cytolytic effector molecules and cytokines [32] that can readily allow the maturation of memory cells. Low doses of IL-2 signaling can support a memory phenotype in activated CD8+ T cells and a T follicular helper-like or memory phenotype in CD4+ T cells. However, the dysregulation or overactivity of IL-2 can have adverse effects, especially when inflammatory disorders [33], immunosuppression, and autoimmunity are involved [34]. Therefore, regulating IL-2 balance is critical. The above isolated compounds were also evaluated for their inhibitory effects on CD3/CD28-induced IL-2 production in Jurkat T cells using different concentrations. Among them, compounds **1** and **2** strongly inhibited IL-2 production at the concentrations tested. Compound **7** significantly suppressed IL-2 production in T cells activated by a co-stimulator. According to the inhibitory effects of these chemicals on IL-2 production, a structural–activity relationship was analyzed. The results revealed that compounds **1**–**4** were furofuran lignans [35,36]. Compounds **2** displayed the strongest inhibition, followed by compound **4**, while compound **3** exhibited a weak inhibitory effect. Thereby, increasing the number of methoxy groups is important for promoting inhibitory effects on IL-2 production in Jurkat T cells induced by a co-stimulator. Additionally, compound **1** showed a ketone group attached furofuran moiety with a strong inhibitory effect on IL-2 production. Thus, this ketone group might affect the activity of furofuran lignans by interacting with nucleophilic sites in proteins and potentially regulating the synthesis or expression of IL-2 production in Jurkat T cells induced by a co-stimulator.

IL-2 is a cytokine that plays a critical role in regulating immune responses, particularly in the activation, proliferation, and differentiation of T cells. Upon binding to IL-2, the receptor undergoes conformational changes and activates downstream signaling pathways, including the MAPK pathway. When the IL-2 receptor is activated, a series of kinases, such as Ras, Raf, MEK, ERK, and MAPK, might be activated, leading to translocation to the nucleus and phosphorylation of various transcription factors involved in the regulation of gene expression and cell growth [37,38]. The phosphorylation of MAPK kinases was activated by a co-stimulator. However, active compounds (**1**, **2**, and **7**) blocked the phosphorylation of ERK/JNK/p38 MAPK, which in turn blocked the downstream effects of these kinases. These compounds may affect immune responses by disrupting the ability of MAPK kinases to participate in immune cell signaling, help control autoimmunity, or prevent excessive inflammation. These active compounds might also modulate immune responses by affecting immune cell activation, differentiation, and cytokine production, offering potential therapeutic effects against cancer, autoimmune diseases, and chronic inflammation.

On the other hand, IL-2 triggers a variety of intracellular signaling cascades when it attaches to IL-2 receptor of T cells. In this process, IL-2 phosphorylates intracellular domains of the receptor by activating several kinases [39]. NFκB is one of the downstream pathways activated by this phosphorylation. On the other hand, the survival and growth of activated T cells are facilitated by NFκB activation, which is crucial for immunological responses [40]. Consequently, the p65 subunit is activated by IL-2 through the NF-κB pathway, which then moves to the nucleus and triggers the transcription of genes involved in immune response control, cell survival, and proliferation. This route is essential for immunological tolerance maintenance and for T cell activity throughout immune responses. The interplay between IL-2 signaling and NF-κB is essential for immune cell expansion and survival as well as for the control of inflammation and immune homeostasis. We found that these active compounds also suppressed the phosphorylation of p65 NFκB, thereby inhibiting the translocation of p65 NFκB from the cytosol to the nucleus. Upon treatment with active compounds **1**, **2**, and **7**, the phosphorylation of IKαβ NFκB was decreased, demonstrating that they might prevent IKαβ degradation. Thus, they can reduce the differentiation of pro-inflammatory T cell subsets (Th1, Th17). Taken together, these results indicate that compounds **1**, **2**, and **7** can influence the biological outcomes of IL-2 signaling by blocking the activation of NFκB and MAPK signaling pathways.

Molecular docking analysis was performed by visualizing the binding pose of each active compound and its interaction with amino acids in the binding pocket of the protein. Compounds **1**, **2**, and **7** docked into the same location with native ligands of ERK, JNK, and p38 proteins, showing significant binding energy. This confirmed their bioactivities revealed by the Western botting assay. On the other hand, compounds **1**, **2**, and **7** docked to the p65 protein. Among them, compound **7** showed the lowest docked score (ΔG = −7.56 kcal/mol). However, compounds **1** and **7** were not present at the binding pose of compound **2** or the reference standard, suggesting that compounds **1** and **7** would not favor binding for the p65 protein. This observation might explain for the stronger suppression by compound **2** than by compounds **1** and **7** toward p65 NFκB expression, as revealed by experimental data.

## 5. Conclusions

In summary, TPC and FBMN-GNPS were assessed to determine the phenolic profiles of extracts and fractions of leaves and twigs of *I. rotunda*. Nine phenolics were isolated and identified from the phenolic profile. The structures of isolated compounds were successfully elucidated. These compounds exhibited significant antioxidative capacity against DPPH and ABTS radicals. They also decreased ROS production in RAW264.7 cells stimulated by LPS in in vitro studies. Isolated compounds also suppressed IL-2 production in Jurkat T cells stimulated by CD3/CD28. Our results demonstrated that zhebeiresinol, syringaresinol, and coniferaldehyde have immune-suppressive effects on T cells by blocking the activation of ERK/JNK/p38 MAPK and p65/IKαβ NFκB. Molecular docking validated the bioactivities of these compounds. Our findings suggest that these compounds are promising against T cell-mediated autoimmune disorders and inflammation.

## Data Availability

All of the data are contained within the article.

## Supplementary Materials

The following supporting information can be downloaded at: https://www.mdpi.com/article/10.3390/antiox14030281/s1. Figure S1. Calibration curve of gallic acid; Figure S2. Total phenolic content of extracts and fraction of leaves and twigs of *I. rotunda*; Figure S3. Interactions of compounds, 1 (Blue), 2 (Magenta), 7 (Green), and KJ4 (N-{3-[(2Z,4S)-1-(2-{[2-(2-amino-1H-imidazol-1-yl)ethyl](methyl)amino}ethyl)-3-(3-cyclohexylpropyl)-2-iminoimidazolidin-4-yl]propyl}guanidine, Cyan), with amino acid when they were docked into ERK protein (PDB ID: 6NBS); Figure S4. Interactions of compounds, **1** (Blue), **2** (Magenta), **7** (Green), and 984 (Cyclopropyl-{4-[5-(3,4-dichlorophenyl)-2-[(1-methyl)-piperidin]-4-yl-3-propyl-3H-imidazol-4-yl]-pyrimidin-2-yl}amine, Cyan), with amino acid when they were docked into JNK protein (PDB ID: 1PMN); Figure S5. Interactions of compounds, **1** (Blue), **2** (Magenta), **7** (Green), and B96 [1-(5-tert-butyl-2-p-tolyl-2H-pyrazol-3-yl)-3-(4-(2-morpholin-4-yl-ethoxy)-naphtalen-1-yl)-urea, Cyan], with amino acid when they were docked into p38 protein (PDB ID: 1KV2); Figure S6. Interactions of compounds, **1** (Blue), **2** (Magenta), **7** (Green), and reference (Cyan), with amino acid when they were docked into p65 protein (PDB ID: 1VKX); Table S1. DPPH and ABTS radical scavenging activities of compounds (**1**, **2**, and **4**).

## References

[1] Tumilaar S.G., Hardianto A., Dohi H., Kurnia D. (2024). A comprehensive review of free radicals, oxidative stress, and antioxidants: Overview, clinical applications, global perspectives, future directions, and mechanisms of antioxidant activity of flavonoid compounds. J. Chem..

[2] Pacher P., Beckman J.S., Liaudet L. (2007). Nitric oxide and peroxynitrite in health and disease. Physiol. Rev..

[3] Genestra M. (2007). Oxyl radicals, redox-sensitive signalling cascades and antioxidants. Cell. Signal..

[4] Mittal M., Siddiqui M.R., Tran K., Reddy S.P., Malik A.B. (2013). Reactive oxygen species in inflammation and tissue injury. Antioxid. Redox Signal..

[5] Maria A.P., Elena P., de Cienfuegos G.A., de Pablo M.A. (2011). Dietary antioxidants: Immunity and host defense. Curr. Top. Med. Chem..

[6] Shields H.J., Traa A., Van Raamsdonk J.M. (2021). Beneficial and detrimental effects of reactive oxygen species on lifespan: A comprehensive review of comparative and experimental studies. Front. Cell Dev. Biol..

[7] Scalbert A., Manach C., Morand C., Rémésy C., Jiménez L. (2005). Dietary polyphenols and the prevention of diseases. Crit. Rev. Food Sci. Nutr..

[8] Matsumura Y., Kitabatake M., Kayano S.-i., Ito T. (2023). Dietary phenolic compounds: Their health benefits and association with the gut microbiota. Antioxidants.

[9] Liu W., Cui X., Zhong Y., Ma R., Liu B., Xia Y. (2023). Phenolic metabolites as therapeutic in inflammation and neoplasms: Molecular pathways explaining their efficacy. Pharmacol. Res..

[10] Ding S., Jiang H., Fang J. (2018). Regulation of immune function by polyphenols. J. Immunol. Res..

[11] Mamun M.A., Rakib A., Mandal M., Kumar S., Singla B., Singh U.P. (2024). Polyphenols: Role in modulating immune function and obesity. Biomolecules.

[12] Pruett S.B., Chambers J.E. (1988). Effects of paraoxon, p-nitrophenol, phenyl saligenin cyclic phosphate, and phenol on the rat interleukin 2 system. Toxicol. Lett..

[13] Zeng W., Cui H., Yang W., Zhao Z. (2022). A systematic review: Botany, phytochemistry, traditional uses, pharmacology, toxicology, quality control and pharmacokinetics of *Ilex rotunda* Thunb. J. Ethnopharmacol..

[14] Peng S., Chen T., Wang G., Li C.-y., Liu W.-j., Wang W.-q., Xuan L.-j. (2023). Five glycosylated phenolic derivatives from the bark of *Ilex rotunda* Thunb. and their anti-inflammatory activities. Nat. Prod. Res..

[15] Cui Y., Zhang Y., Liu G. (2015). Syringin may exert sleep-potentiating effects through the NOS/NO pathway. Fundam. Clin. Pharmacol..

[16] Le D.D., Jang Y.S., Truong V., Dinh T., Dang T., Yu S., Lee M. (2024). Anti-inflammatory effects and metabolomic analysis of *Ilex rotunda* extracted by supercritical fluid extraction. Int. J. Mol. Sci..

[17] Le D.D., Yu S., Dang T., Lee M. (2023). Molecular networking and bioassay-guided preparation and separation of active extract and constituents from *Vicia tenuifolia* Roth. Antioxidants.

[18] Zhu B.-R., Pu S.-B., Wang K.D.G., Xu D.-R., Zhou H.-H. (2013). Chemical constituents of the aerial part of *Gynura segetum*. Biochem. Syst. Ecol..

[19] Ouyang M.-A., Wein Y.-S., Zhang Z.-K., Kuo Y.-H. (2007). Inhibitory activity against Tobacco Mosaic Virus (TMV) replication of pinoresinol and syringaresinol lignans and their glycosides from the root of *Rhus javanica* var. roxburghiana. J. Agric. Food Chem..

[20] Lee J.W., Lee J.H., Lee C., Jin Q., Lee D., Kim Y., Hong J.T., Lee M.K., Hwang B.Y. (2015). Inhibitory constituents of *Sophora tonkinensis* on nitric oxide production in RAW 264.7 macrophages. Bioorganic Med. Chem. Lett..

[21] Wu Y., Su J., Guo R.-x., Ren T.-k., Zhang M., Dong M., Sauriol F., Shi Q., Gu Y., Huo C. (2014). Two new non-taxoids from leaves of *Taxus cuspidata*. Chem. Nat. Compd..

[22] Fang J.-M., Wei K.-M., Cheng Y.-S. (1985). A study of the constituents of the heartwood of *Tsuga Chinensis Pritz. Var. Formosana* (Hay.). J. Chin. Chem. Soc..

[23] Kalikar R.G., Deshpande R.S., Chandalia S.B. (1986). Synthesis of vanillin and 4-hydroxybenzaldehyde by a reaction scheme involving condensation of phenols with glyoxylic acid. J. Chem. Technol. Biotechnol..

[24] Cho J.-Y., Moon J.-H., Seong K.-Y., Park K.-H. (1998). Antimicrobial activity of 4-hydroxybenzoic acid and *trans* 4-hydroxycinnamic Acid Isolated and Identified from Rice Hull. Biosci. Biotechnol. Biochem..

[25] Gulcin İ., Alwasel S.H. (2023). DPPH Radical Scavenging Assay. Processes.

[26] Liu T., Zhang L., Joo D., Sun S.-C. (2017). NF-κB signaling in inflammation. Signal Transduct. Target. Ther..

[27] Suriya U., Mahalapbutr P., Rungrotmongkol T. (2022). Integration of in silico strategies for drug repositioning towards P38α mitogen-activated protein kinase (MAPK) at the allosteric Site. Pharmaceutics.

[28] Chen F.E., Huang D.-B., Chen Y.-Q., Ghosh G. (1998). Crystal structure of p50/p65 heterodimer of transcription factor NF-κB bound to DNA. Nature.

[29] Chen J., Yang J., Ma L., Li J., Shahzad N., Kim C.K. (2020). Structure-antioxidant activity relationship of methoxy, phenolic hydroxyl, and carboxylic acid groups of phenolic acids. Sci. Rep..

[30] Bhol N.K., Bhanjadeo M.M., Singh A.K., Dash U.C., Ojha R.R., Majhi S., Duttaroy A.K., Jena A.B. (2024). The interplay between cytokines, inflammation, and antioxidants: Mechanistic insights and therapeutic potentials of various antioxidants and anti-cytokine compounds. Biomed. Pharmacother..

[31] Yang Y., Bazhin A.V., Werner J., Karakhanova S. (2013). Reactive oxygen species in the immune system. Int. Rev. Immunol..

[32] Ross S.H., Cantrell D.A. (2018). Signaling and function of interleukin-2 in T lymphocytes. Annu. Rev. Immunol..

[33] Lykhopiy V., Malviya V., Humblet-Baron S., Schlenner S.M. (2023). IL-2 immunotherapy for targeting regulatory T cells in autoimmunity. Genes Immun..

[34] Orozco Valencia A., Camargo Knirsch M., Suavinho Ferro E., Antonio Stephano M. (2020). Interleukin-2 as immunotherapeutic in the autoimmune diseases. Int. Immunopharmacol..

[35] Chi Y., He H.-W., Chen C.-Y., Zhao S.-Y., Zhou H., Xu D., Liu X., Xu G. (2023). Furofuran lignans for plant protection: Discovery of sesamolin and its derivatives as novel anti-tobacco Mosaic Virus and antibacterial agents. J. Agric. Food Chem..

[36] Xu W.-H., Zhao P., Wang M., Liang Q. (2019). Naturally occurring furofuran lignans: Structural diversity and biological activities. Nat. Prod. Res..

[37] Xu W., Yan M., Lu L., Sun L., Theze J., Zheng Z., Liu X. (2001). The p38 MAPK pathway is involved in the IL-2 induction of TNF-β gene via the EBS element. Biochem. Biophys. Res. Commun..

[38] Koike T., Yamagishi H., Hatanaka Y., Fukushima A., Chang J.-w., Xia Y., Fields M., Chandler P., Iwashima M. (2003). A novel ERK-dependent signaling process that regulates interleukin-2 expression in a late phase of T cell activation. J. Biol. Chem..

[39] Minami Y., Taniguchi T. (1995). IL-2 signaling: Recruitment and activation of multiple protein tyrosine kinases by the components of the IL-2 receptor. Curr. Opin. Cell Biol..

[40] Pimentel-Muiños F.X., Mazana J., Fresno M. (1994). Regulation of interleukin-2 receptor alpha chain expression and nuclear factor.kappa B activation by protein kinase C in T lymphocytes. Autocrine role of tumor necrosis factor alpha. J. Biol. Chem..

