# Supplementary material for: Phenolics from Ilex rotunda Possess Antioxidative Effects and Block Activation of MAPK and NF-κB Signaling by Inhibiting IL-2 Production in CD3/CD28 Activated Jurkat T Cells
Source: Antioxidants (Basel). 2025 Feb 27;14(3):281. doi: 10.3390/antiox14030281 (PMC11939502; doi:10.3390/antiox14030281)
Supplement: Supplementary file 1 [file antioxidants-14-00281-s001.zip › antioxidants-3484258-supplementary.pdf]

Supplementary materials

Phenolics from *Ilex rotunda* possess antioxidative effects and block activation of MAPK and NF-κB signaling by inhibiting IL-2 production in CD3/CD8 activated Jurkat T cells

DucDat Le[1], Vinhquang Truong[1], Thinhulinh Dang[1], Soojung Yu[2], Thientam Dinh[1] and Mina Lee[1,2,*]

[1]College of Pharmacy and Research Institute of Life and Pharmaceutical Sciences, Sunchon National University, 255 Jungangno, Suncheon 57922, Jeonnam, Republic of Korea

[2] Department of Natural Cosmetics Science and Natural Cosmetics Research Institute, Sunchon National University, 255 Jungangno, Suncheon 57922, Jeonnam, Republic of Korea.

*Correspondence: minalee@sunchon.ac.kr/ minalee@scnu.ac.kr; Tel.: +82-61-750-3764; Fax: +82-61-750-3708

Table of Contents

Figure S1. Calibration curve of gallic acid 1

Figure S2. Total phenolic content of extracts and fraction of leaves and twigs of *I. rotunda* 1

Table S1. DPPH and ABTS radical scavenging activities of compounds (**1**, **2**, and **4**)....................2

Figure S3. Interactions of compounds, **1** (Blue), **2** (Magenta), **7** (Green), and KJ4 (N-{3-[(2Z,4S)-1-(2-{[2-(2-amino-1H-imidazol-1-yl)ethyl](methyl)amino}ethyl)-3-(3-cyclohexylpropyl)-2-iminoimidazolidin-4-yl]propyl}guanidine, Cyan), with amino acid when they were docked into ERK protein (PDB ID: 6NBS)....................2

Figure S4. Interactions of compounds, **1** (Blue), **2** (Magenta), **7** (Green), and 984 (Cyclopropyl-{4-[5-(3,4-dichlorophenyl)-2-[(1-methyl)-piperidin]-4-yl-3-propyl-3H-imidazol-4-yl]-pyrimidin-2-yl}amine, Cyan), with amino acid when they were docked into JNK protein (PDB ID: 1PMN)......3

Figure S5. Interactions of compounds, **1** (Blue), **2** (Magenta), **7** (Green), and B96 [1-(5-tert-butyl-2-p-tolyl-2H-pyrazol-3-yl)-3-(4-(2-morpholin-4-yl-ethoxy)-naphtalen-1-yl)-urea, Cyan], with amino acid when they were docked into p38 protein (PDB ID: 1KV2)....................4

Figure S6. Interactions of compounds, **1** (Blue), **2** (Magenta), **7** (Green), and reference (Cyan), with amino acid when they were docked into p65 protein (PDB ID: 1VKX)....................5

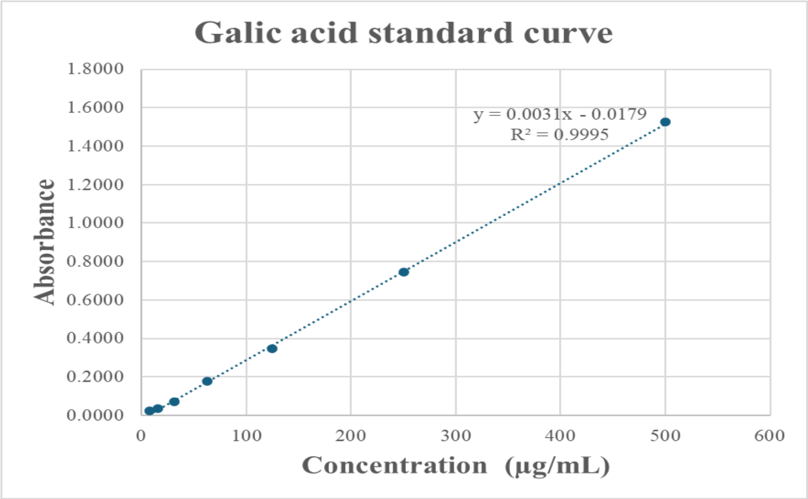

Figure S1. Calibration curve of gallic acid.

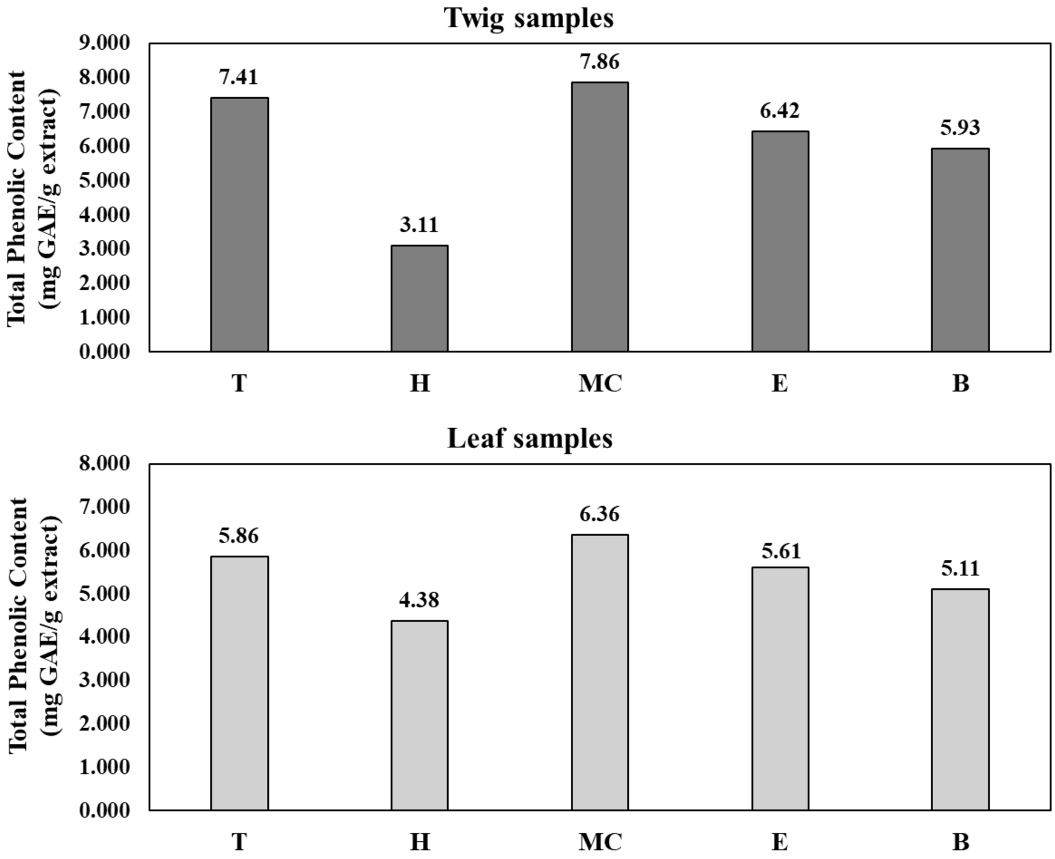

Figure S2. Total phenolic content of extracts and fraction of leaves and twigs of *I. rotunda*.

Table S1. DPPH and ABTS radical scavenging activities of compounds (**1**, **2**, and **4**).

**Compound**	**EC_{50} (μM)**	
	**DPPH**	**ABTS**
**1**	39.16 ± 1.04	33.23 ± 0.66
**2**	9.72 ± 1.99	42.90 ± 2.12
**4**	70.73 ± 9.21	57.62 ± 6.00
**Ascorbic acid (AA)**	4.22 ± 0.17	24.83 ± 0.27

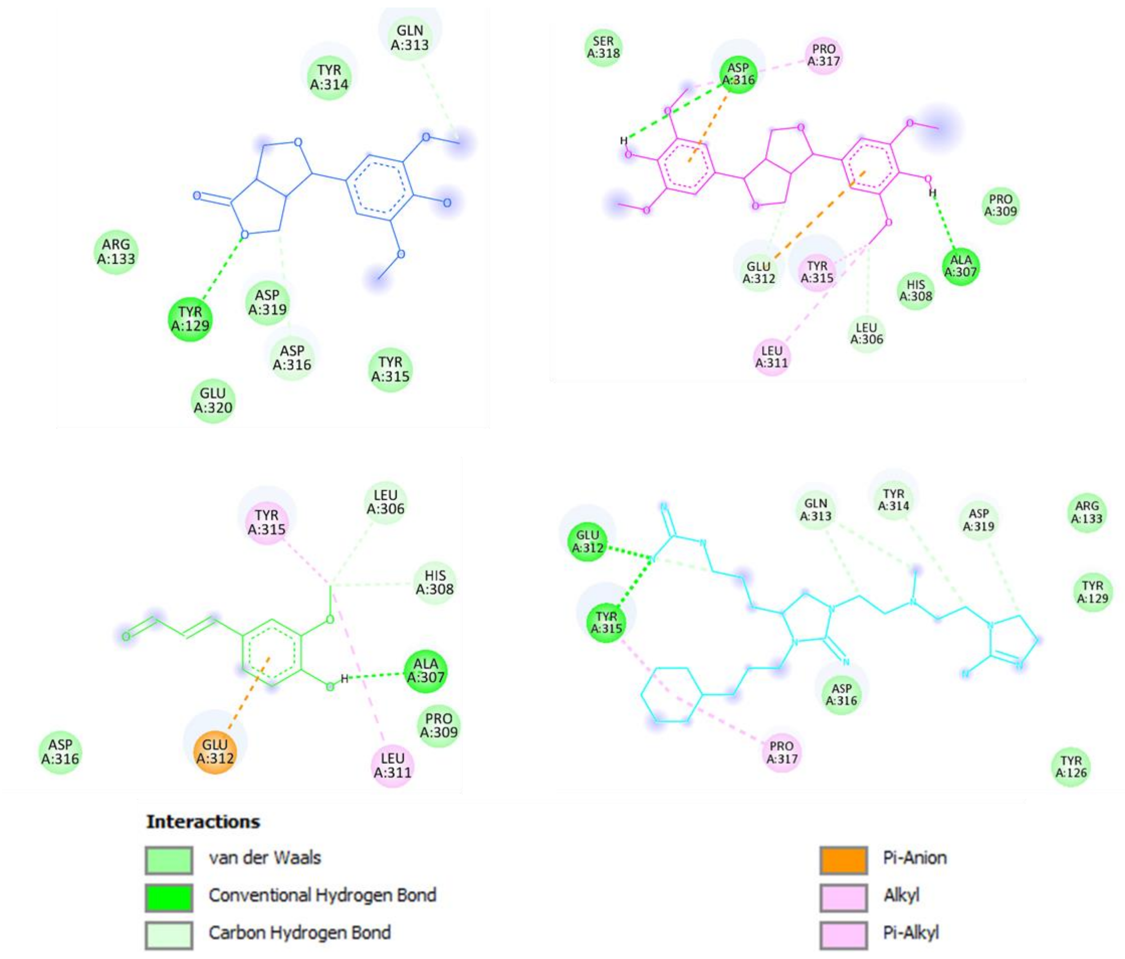

Figure S3. Interactions of compounds, **1** (Blue), **2** (Magenta), **7** (Green), and KJ4 (N-{3-[(2Z,4S)-1-(2-{[2-(2-amino-1H-imidazol-1-yl)ethyl](methyl)amino}ethyl)-3-(3-cyclohexylpropyl)-2-iminoimidazolidin-4-yl]propyl}guanidine, Cyan), with amino acid when they were docked into ERK protein (PDB ID: 6NBS).

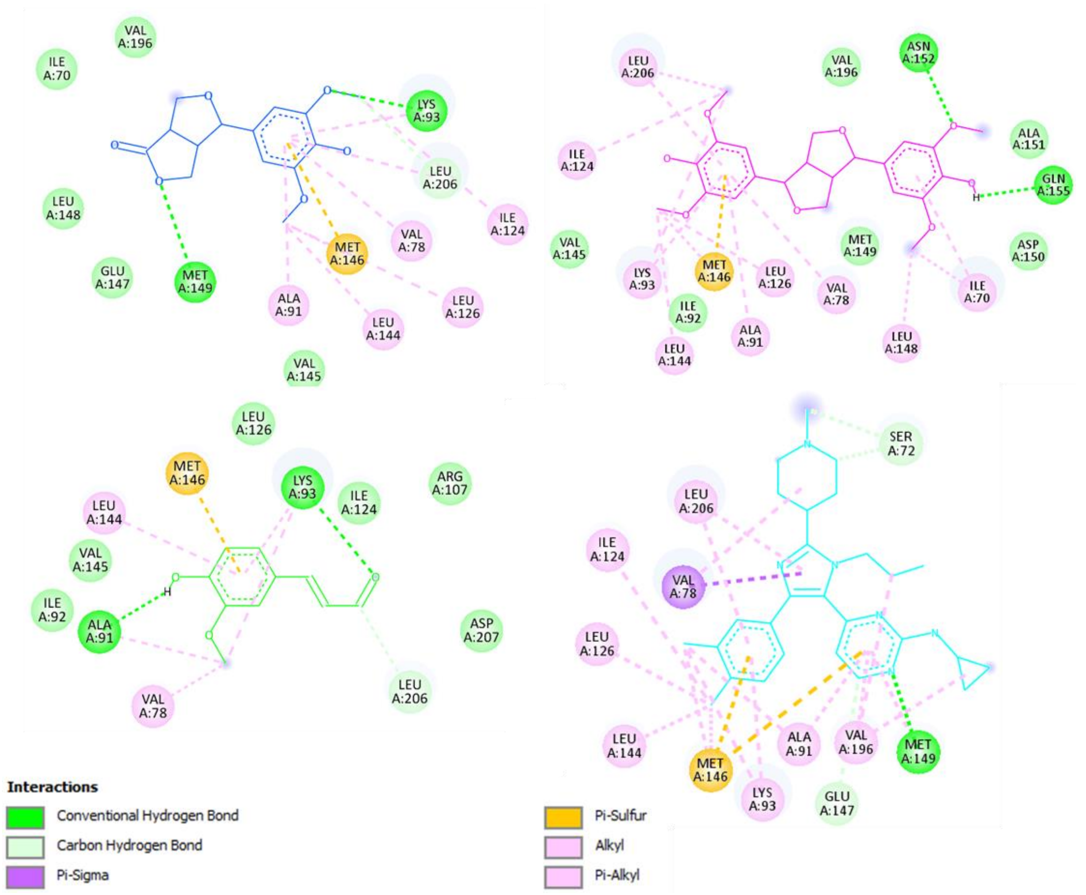

Figure S4. Interactions of compounds, **1** (Blue), **2** (Magenta), **7** (Green), and 984 (Cyclopropyl-{4-[5-(3,4-dichlorophenyl)-2-[(1-methyl)-piperidin]-4-yl-3-propyl-3H-imidazol-4-yl]-pyrimidin-2-yl}amine, Cyan), with amino acid when they were docked into JNK protein (PDB ID: 1PMN).

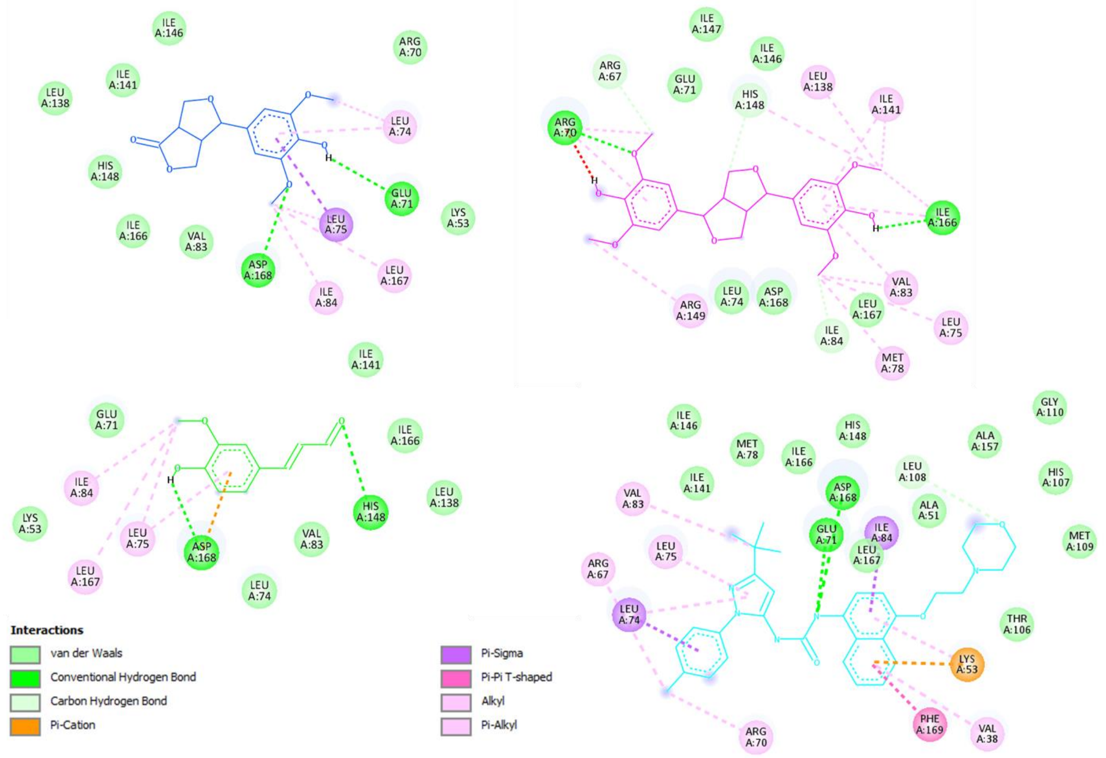

Figure S5. Interactions of compounds, **1** (Blue), **2** (Magenta), **7** (Green), and B96 [1-(5-tert-butyl-2-p-tolyl-2H-pyrazol-3-yl)-3-(4-(2-morpholin-4-yl-ethoxy)-naphtalen-1-yl)-urea, Cyan], with amino acid when they were docked into p38 protein (PDB ID: 1KV2).

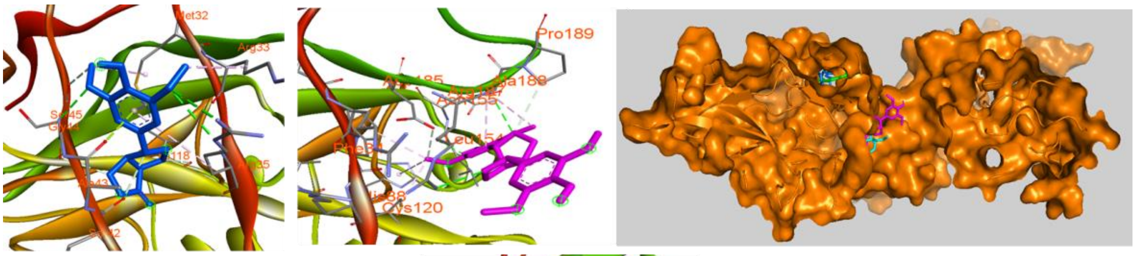

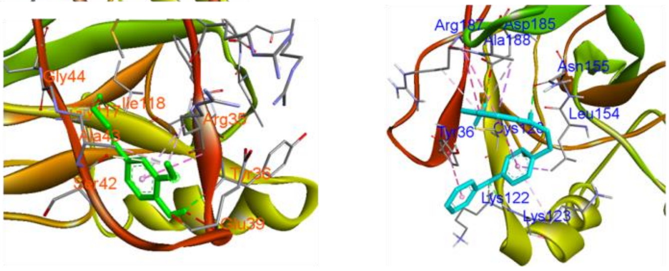

Ligands	Scores (kcal/mol)
**1**	**–4.40**
**2**	**–5.81**
**7**	**–7.56**
**Reference**	**–7.18**

p65 protein (PDB ID: 1VKX)

Figure S6. Interactions of compounds, **1** (Blue), **2** (Magenta), **7** (Green), and reference (Cyan), with amino acid when they were docked into p65 protein (PDB ID: 1VKX).
